# Wafer-Scale Vertical 1D GaN Nanorods/2D MoS_2_/PEDOT:PSS for Piezophototronic Effect-Enhanced Self-Powered Flexible Photodetectors

**DOI:** 10.1007/s40820-024-01553-8

**Published:** 2024-11-05

**Authors:** Xin Tang, Hongsheng Jiang, Zhengliang Lin, Xuan Wang, Wenliang Wang, Guoqiang Li

**Affiliations:** 1 State Key Laboratory of Luminescent Materials and Devices, South China University of Technology, Guangzhou, 510640 People’s Republic of China; 2 Department of Electronic Materials, School of Materials Science and Engineering, South China University of Technology, Guangzhou, 510640 People’s Republic of China

**Keywords:** Vertical nanorod arrays, van der Waals heterostructure, Piezophototronic effect, Self-powered photodetection, Flexible sensors

## Abstract

van der Waals (vdW) heterostructures constructed by low-dimensional (0D, 1D, and 2D) materials are emerging as one of the most appealing systems in next-generation flexible photodetection. Currently, hand-stacked vdW-type photodetectors are not compatible with large-area-array fabrication and show unimpressive performance in self-powered mode. Herein, vertical 1D GaN nanorods arrays (NRAs)/2D MoS_2_/PEDOT:PSS in wafer scale have been proposed for self-powered flexible photodetectors arrays firstly. The as-integrated device without external bias under weak UV illumination exhibits a competitive responsivity of 1.47 A W^−1^ and a high detectivity of 1.2 × 10^11^ Jones, as well as a fast response speed of 54/71 µs, thanks to the strong light absorption of GaN NRAs and the efficient photogenerated carrier separation in type-II heterojunction. Notably, the strain-tunable photodetection performances of device have been demonstrated. Impressively, the device at − 0.78% strain and zero bias reveals a significantly enhanced photoresponse with a responsivity of 2.47 A W^−1^, a detectivity of 2.6 × 10^11^ Jones, and response times of 40/45 µs, which are superior to the state-of-the-art self-powered flexible photodetectors. This work presents a valuable avenue to prepare tunable vdWs heterostructures for self-powered flexible photodetection, which performs well in flexible sensors.

**Supplementary Information:**

The online version contains supplementary material available at 10.1007/s40820-024-01553-8.

## Highlights

- Vertical 1D GaN nanorod arrays/2D MoS_2_/PEDOT:PSS heterostructures in wafer scale have been fabricated for flexible photodetection firstly.
- Self-powered flexible photodetector at compressive strain reveals a significantly enhanced photoresponse with a responsivity of 2.47 A W^−1^ and response times of 40/45 µs, which are superior to the state-of-the-art flexible devices.
- This work not only provides a valuable strategy for the design and construction of tunable van der Waals heterostructures, but also opens a new opportunity for flexible sensors.

**Supplementary Information:**

The online version contains supplementary material available at 10.1007/s40820-024-01553-8.

## Graphical abstract

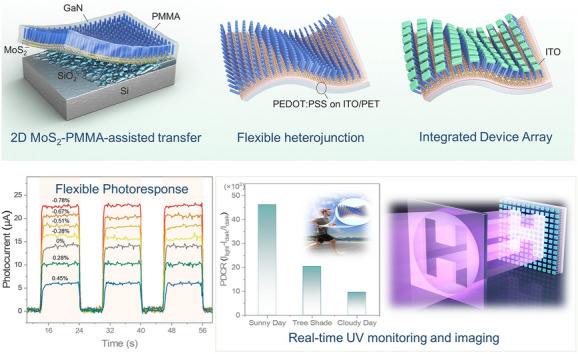

## Introduction

Flexible photodetectors, as an essential component of sensor networks, have attracted enormous attention for their extensive application in health monitoring, motion detection, biomedical imaging, and foldable display [1–4]. Currently, the operation of flexible photodetectors requires external power supply, thereby greatly increasing the size, weight, and energy consumption of the detection system and limiting the development of miniaturized and lightweight portable equipment [4–6]. Recently, self-powered devices have been demonstrated to be constructive in next-generation sensors [7–13]. In general, most reported self-powered technology can be achieved by constructing Schottky junction [7, 8], p–n junction [9, 10] or heterojunction [11–13]. Among them, heterojunction-type photodiodes constructed of semiconductors with different Fermi energy levels can produce build-in electric field to drive the photogenerated charge flow, providing engineering flexibility, which has stimulated the research enthusiasm for emerging functional semiconductors that are bendable [14, 15].

Over recent years, various functional materials for constructing flexible heterostructures have been explored, such as two-, one-, and zero-dimensional (2D, 1D and 0D) materials [16, 17] as well as perovskite [18, 19]. Considering performance degradation of perovskite film-based devices in long-term operation [19, 20], van der Waals (vdW) heterostructures constructed by low-dimensional materials with excellent mechanical capability, fascinating physical properties, and high transparency are highly desirable for flexible electronics/optoelectronics [21, 22]. Benefiting from the dangling bond-free surfaces of 2D materials, vdW integration enables the assembly of diverse materials into hybrid system by manual stacking for self-powered, flexible photodetection without considering strict lattice mismatching, such as 2D Ga_2_O_3_/2D PbI_2_ [22], 2D WS_2_/2D Graphene [23], 1D ZnO/2D WSe_2_ [24], 1D GaAs/2D WSe_2_ [25], and 0D Bi/2D C_3_N_4_ [26]. However, the weak light absorption of ultrathin 2D material results in poor photoresponse [22–26], where responsivity performances, describing the photoelectric conversion capability of devices, are mostly in the order of μA W^−1^. In addition, the large-area array design and fabrication of vdW devices still remain a significant challenge, greatly limiting its mass production and practical application [27–29].

As an emerging technology, vertical heterostructures incorporating 1D nanorod arrays (NRAs) assembled on 2D materials provide a promising pathway to develop self-powered photodetectors arrays with outstanding photoresponse capacity, which can be ascribed to the strong light absorption in nanoarray structures with huge surface-to-volume ratio [8, 11]. In the latest research activities, vdW epitaxy of GaN NRAs on 2D materials such as graphene [30], Mxene [31] and transition metal dichalcogenides (TMDs) (e.g., MoS_2_, WS_2_, and MoSe_2_) [32] on Si substrates has been investigated for optoelectronic device. Remarkably, GaN NRAs/MoS_2_/Si photodetectors at zero bias exhibit record responsivity over 10 A W^−1^ among vdW device. These integrated systems are based on a 1D/2D structure as an absorber layer and Si substrates as a hole transport layer (HTL) to collect charge carriers. Nevertheless, rigid Si-based integrated systems are not suitable for preparing flexible devices. Instead, poly(3,4-ethylenedioxythiophene):poly (styrenesulfonate) (PEDOT:PSS) as an efficient HTL material has been widely studied in flexible photovoltaic devices [33] and its combination with GaN has been proved to improve self-powered photodetection performance [34, 35]. However, traditional flexible materials with poor thermal stability can hardly endure the high growth temperatures of GaN and TMDs [36], and thus, their 1D/2D heterojunctions integrated with PEDOT:PSS for flexible optoelectronics have not been previously reported. Furthermore, the piezoelectric effects of wurtzite structured GaN NRAs in bending state on the photoresponse of hybrid systems have not been well understood.

In this work, wafer-scale 1D GaN NRAs/2D MoS_2_/PEDOT:PSS heterostructures have been demonstrated for self-powered flexible photodetectors arrays firstly. The atomically thin MoS_2_ with excellent mechanical strength and physicochemical stability not only serves as a template for GaN NRAs vdW epitaxy, but also enables them to be transferred onto flexible PEDOT:PSS/substrate losslessly, which presents a promising avenue for functional integration and large-area fabrication of flexible electronics/optoelectronics. Benefitting from the enhanced photogenerated current density on account of the strong light absorption of nanoarray structures, a high light on/off ratio above 1.2 × 10^5^ of 1D/2D hybrid system has been demonstrated. Accordingly, the integrated device, without external bias, under weak UV illumination exhibits a competitive responsivity of 1.47 A W^−1^ and a detectivity of 1.2 × 10^11^ Jones, as well as a fast rise/decay times of 54/71 µs, which is attributed to the efficient carrier separation in type-II heterojunction. Besides, the strain-tunable photodetection performance of devices has been demonstrated. The energy band tilt at the GaN/MoS_2_ interface originating from strain-induced piezopolarization charges promotes the photogenerated carrier separation and transport. Impressively, the device at − 0.78% strain reveals a significantly enhanced photoresponse with a responsivity of 2.47 A W^−1^, a detectivity of 2.6 × 10^11^ Jones, and response times of 40/45 µs, which are superior to the state-of-the-art self-powered flexible photodetectors. Furthermore, the prepared photodetectors perform well in real-time UV monitoring and imaging. This work not only provides a valuable strategy for the design and construction of tunable vdW heterostructures, but also opens a new opportunity for flexible sensors.

## Experimental Section

**Fig. 1:**
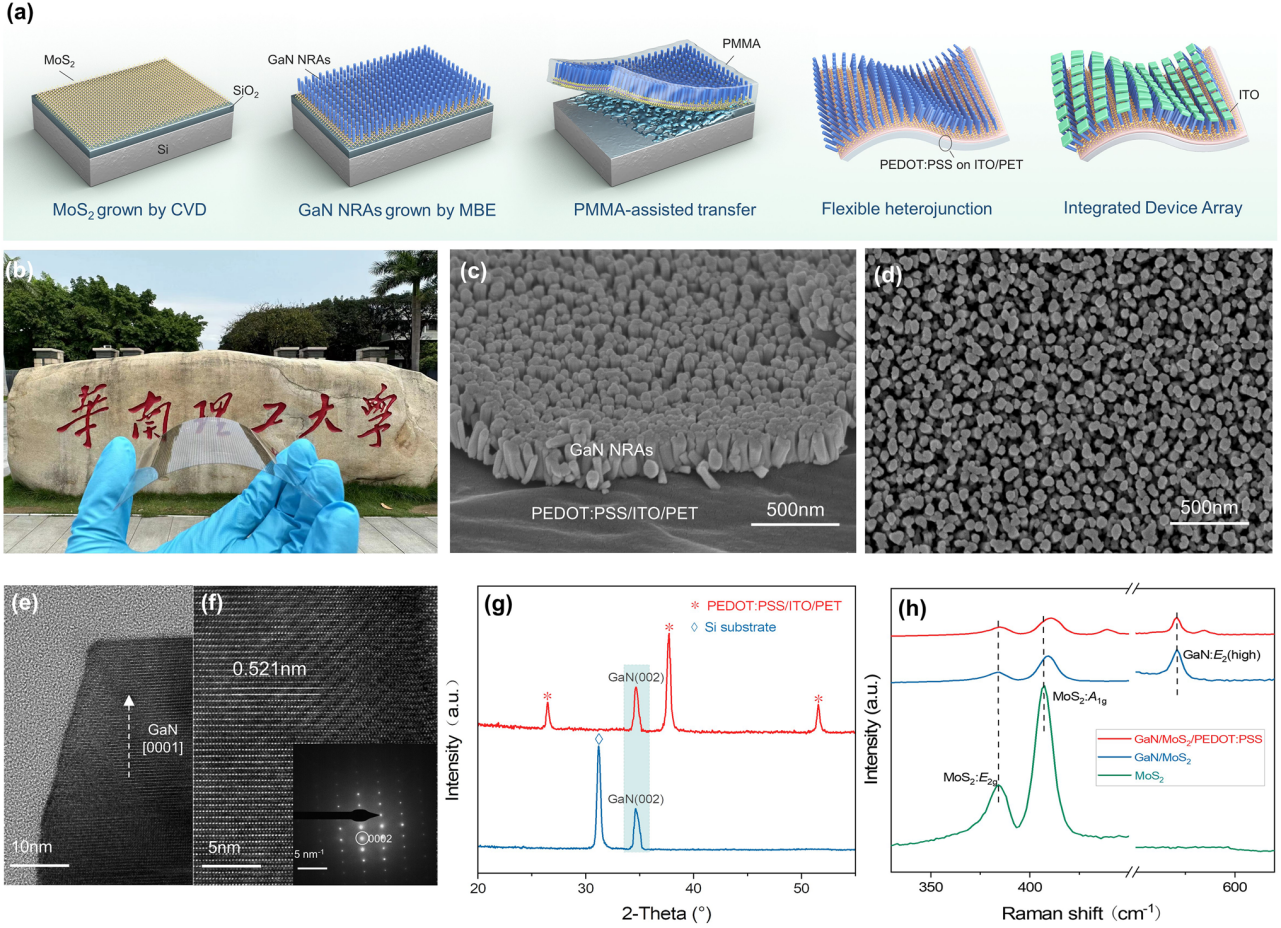
Fabrication process and characterization of GaN NRAs/MoS_2_/PEDOT:PSS heterojunction-based photodetector. **a** Schematic illustration of fabrication process of vertical heterostructures for flexible photodetector arrays. **b** Optical image of the heterojunctions in front of Canton Tower. **c** Oblique-view and **d** top-view SEM images of heterojunctions. **e** TEM image of GaN NR, where the white-dotted arrow points in the direction of growth, and **f** its high-resolution image with the corresponding SAED patterns. **g** XRD patterns and **h** Raman spectrum of GaN NRAs/MoS_2_ assembled on PEDOT:PSS/ITO/PET (red) and SiO_2_/Si (blue), pristine MoS_2_ as references (green). (Color figure online)

Vertical GaN NRAs/MoS_2_/PEDOT:PSS heterostructures in wafer scale have been fabricated firstly by combining vdW epitaxy with wet transfer, which is described in Fig. 1a. Detailed information can be found in Fig. S1.

### Materials

SiO_2_/Si wafers, sulfur (S) powder, polyethylene terephthalate (PET), and PEDOT:PSS were supplied by Shanghai PrMat Co., Ltd. (China). Molybdenum (Mo) metal and gallium (Ga) particle were purchased by Beijing Zhongnuo Co., Ltd. (China). Hydrofluoric acid (HF, 35 wt%), acetone, and ethyl alcohol were supplied from Sinopharm Chemical Reagent Co., Ltd. (China).

### Device Fabrication

Wafer-scale MoS_2_ films were grown on SiO_2_/Si substrates by using chemical vapor deposition (CVD) [37]. The precursor Mo was deposited onto 2-inch SiO_2_/Si substrates by magnetron sputtering technology at 10 W power for 10 s. Then MoS_2_ films were synthesized by sulfurization in a dual-temperature zone tube furnace. Substrate and 0.1 g S powder were set at 660 and 180 °C for 1 h. GaN NRAs were epitaxially grown on MoS_2_/SiO_2_/Si substrates through plasma-assisted MBE (PA-MBE) by Ga droplet-assisted method using plasma-assisted MBE (MANTIS) [32]. The growth process can be divided into two steps: 180 s of Ga droplet pre-deposition and 180 min of growth of nanorods as followed. The substrate temperature and rotation speed were set as 760 °C and 2 rpm, respectively. The metal flux of Ga was detected as 1.3 ~ 1.4 × 10^–7^ Torr. The N_2_ flux and the corresponding forward plasma power were set as 1.2 sccm and 400 W, respectively. GaN NRAs/MoS_2_ hybrid system was transferred losslessly onto a PEDOT:PSS/ITO/PET substrate for flexible heterojunctions by poly(methyl methacrylate) (PMMA)-assisted wet etching method [38]. Firstly, the PMMA was spin-coated onto GaN NRAs/MoS_2_ at 2000 r min^−1^. Secondly, the SiO_2_ substrate was etched with 5% solution of HF until GaN NRAs/MoS_2_ was detached. PEDOT:PSS was mixed with isopropyl alcohol in a volume ratio of 1:2 and then spin-coated on ITO/PET substrate. The floating PMMA-coated GaN NRAs/MoS_2_ was transferred onto PEDOT:PSS/ITO/PET flexible substrate. After drying, the PMMA on the top of GaN NRAs was removed by oxygen plasma etching. Eventually, 120-nm ITO electrode (1 mm^2^) was deposited according to the mask on the head of sample to integrate the nanorods for photodetector array.

### Heterojunction Characterization

The morphologies of samples were investigated by field-emission SEM (Hitachi SU-8020) attached with EDS (HORIBA EX-350) system. Structural properties were studied by HRTEM (FEI Talos F200X) and XRD (Bruker D8 ADVANCE). Material phase was determination by micro-Raman spectroscopy (Renishaw inVia) using a linear polarized 532-nm laser. XPS (Thermal ESCALAB 250Xi) was employed to confirm the composition transitions. The strain effect on interfacial carrier behaviors of heterojunction was profoundly studied by room-temperature PL (MStarter 100) under 532 nm laser excitation. The band structures of GaN/MoS_2_/PEDOT:PSS were analyzed by XPS valence band edge and UPS (Thermo ESCALAB 250Xi). The charge transfer and separation in heterojunctions were determined by FS-TAS (Ultrafast System HELIOS) with an excitation source of 350 nm and a detection range of 450–750 nm. All calculations based on DFT were performed based on Cambridge Sequential Total Energy Package (CASTEP) code of Material Studio calibrated by Perdew–Burke–Ernzerhof (PBE) and generalized gradient approximation (GGA). The Monkhorst–Pack k-point of 4 × 4 × 2 and the energy cutoff of 381 eV were set.

### Device Measurement

The optoelectronic performances of photodetectors were measured using a semiconductor device analyzer (KEYSIGHT B1505A), and a LED with adjustable power density was used as UV light source. The time responses were recorded by a digital oscilloscope (Tektronix MDO 3102) under UV light chopped by an optical chopper. The outdoor tests occurred in front of Canton Tower (Sunny day, October 11, 2023), Sun Yat-sen statue (Tree shade, October 11, 2023) and square (Cloudy, December 20, 2023) at South China University of Technology.

## Results and Discussion

### Characterization of Heterostructures

The as-prepared GaN NRAs/MoS_2_/PEDOT:PSS heterojunctions, in Fig. S2, show more than 55% transparency in the visible wavelength range (400–800 nm), which is constructive for flexible transparent electronics/optoelectronics. Figure 1b displays an optical image of GaN/MoS_2_/PEDOT:PSS device array with high transparency and flexibility, at the gate of South China University of Technology. The morphology of GaN NRAs/MoS_2_/PEDOT:PSS heterostructures was characterized by top-view and oblique-view SEM in Fig. 1c, d, depicting that GaN NRAs with lengths of ~ 500 nm and diameters of ~ 50 nm are arranged almost vertically on flexible substrate. The single-crystal structure of GaN NRAs was further investigated by the HRTEM images and their corresponding SAED pattern, as shown in Fig. 1e, f. The images show an interplanar spacing of ∼0.521 nm derived in the axial direction, consistent well with the wurtzite GaN structure as reported [38–40]. As shown in X-ray diffraction (XRD) pattern, Fig. 1g, strong diffraction peaks at 34.4° can be attributed to (0002) plane of the hexagonal GaN structure (JCPDS No. 50-0792), confirming that vertically aligned GaN NRAs with c-axis orientation have been transferred onto a flexible substrate [41]. Furthermore, Raman spectrums were employed to study the bare MoS_2_, GaN/MoS_2_ and GaN/MoS_2_/PEDOT:PSS, described in Fig. 1h. The peaks difference between in-plane vibration mode (*E*_1g_) and out-of-plane vibrations mode (*A*_1g_) of MoS_2_ was identified as 23 cm^−1^, indicating a 3L MoS_2_ contiguous film [39, 42]. It is worth noting that the *A*_1g_ phonon mode of heterojunctions exhibits a significant blue shift compared with bare MoS_2_, suggesting a strong interfacial interaction [43, 44]. For self-assembly heterojunctions, the additional peak at 569 cm^−1^ can be attributed to the GaN *E*_2_ (high) phonon mode, and the peaks of MoS_2_ are also observed, indicating no phase transition in MoS_2_ after GaN growth [45]. The GaN *E*_*2*_ (high) is very sensitive to biaxial strain and thus is extensively used to characterize the in-plane stress state of the GaN [46]. In Fig. S3, it reveals a significant redshift for the phonon peak with gradually increasing the applied tensile strain. On the contrary, the peak appears blue shift when the compressive strain is applied. The binding information and composition of GaN/MoS_2_/PEDOT:PSS and MoS_2_/SiO_2_/Si (as references) were determined by X-ray photoelectron spectroscopy (XPS), as shown in Fig. S4. The main peaks assigned to Mo 3*d*_3/2_, Mo 3*d*_5/2_, S 2*p*_1/2_, and S 2*p*_3/2_ were identified in the fitted XPS spectra of both samples, demonstrating MoS_2_ still exists after the epitaxy and transfer of GaN NRAs [47]. Remarkably, Mo 3*d* and S 2*p* peaks in GaN/MoS_2_/PEDOT:PSS heterojunction shows a significant upshift compared to that of bare MoS_2_, which may be attributed to the migration of electrons from MoS_2_ to GaN, as previously reported [47, 48]. The corresponding broadened peaks reflect the quality of MoS_2_ has been affected by the epitaxial growth and transfer process [49].

### Charge Transport at GaN NRAs/MoS_2_/PEDOT:PSS Heterointerfaces

**Fig. 2:**
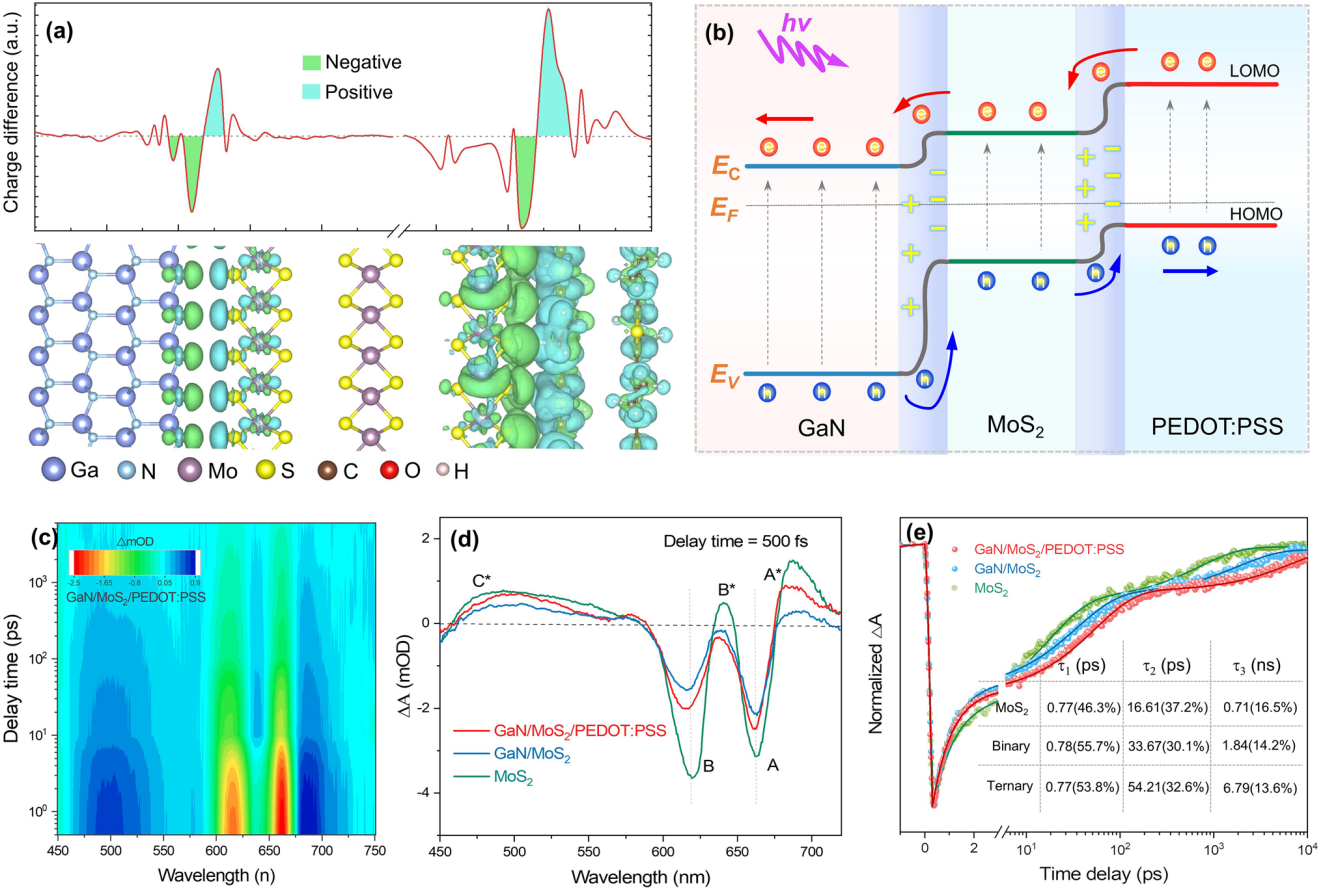
Investigation for the photogenerated charge transport of GaN NRAs/MoS_2_/PEDOT:PSS. **a** Proposed type-II band alignment diagrams of the GaN/MoS_2_/PEDOT:PSS heterojunction. **b** Calculated charge density difference (CDD) isosurface distributions and planar-averaged CDD along the *Z* position along vertical heterojunction. **c** Transient absorption (TA) mapping of GaN/MoS_2_/PEDOT:PSS at different indicated time delays under 350 nm excitation. **d** Milli-optical density (mOD) of MoS_2_, GaN/MoS_2_, and GaN/MoS_2_/PEDOT:PSS heterojunctions at 500 fs. **e** The normalized kinetics of A excitons extracted from **d**

To clarify the photogenerated charge transport at heterointerfaces, the band alignment of GaN/MoS_2_/PEDOT:PSS was analyzed theoretically and experimentally. As shown in Fig. S5, the bandgaps (*E*_g_) of PEDOT:PSS, MoS_2_, and GaN were estimated as 3.0, 1.8, and 3.4 eV. Accordingly, the work functions (Φ) were calculated as 4.21, 4.69, and 5.05 eV, respectively [50]. Besides, the energy difference between the Fermi level and the highest occupied molecular orbital (HOMO)/valence band maximum (VBM) for PEDOT:PSS, MoS_2_, and GaN was determined as 1.12, 0.95, and 2.43 eV, respectively [51]. The local density of states (LDOS) for heterojunction were calculated by using density functional theory (DFT) in Fig. S6, which further confirms it. Furthermore, the heterointerface charge transfer of GaN/MoS_2_/PEDOT:PSS was investigated by calculating the charge density difference (CDD) distribution, in Fig. 2a. As illustrated in the isosurface diagram, the green region is the charge accumulation region, and the blue region means the charge depletion region, corresponding to the positive and negative values in the planar-averaged CDD along the Z position, respectively. Based on the above results, the band diagram of GaN/MoS_2_/PEDOT:PSS is constructed in Fig. 2b, showing the type-II band alignment at the heterointerface, which facilitates carrier separation in self-powered mode [52].

The photoexcited carrier dynamics at GaN/MoS_2_/PEDOT:PSS heterointerface were investigated by femtosecond transient absorption (FS-TA) spectroscopy. The TA spectra of the excited heterojunction at different delays are shown in Fig. 2c, where the main negative signals A, B are attributed to the ground-state bleaching (GSB) due to state filling or Pauli blocking of the excitonic resonances [53, 54]. Ultrafast dynamic spectroscopy for MoS_2_ and GaN/MoS_2_ was studied under the same condition in Fig. S7. At pump density (50 μJ cm^−2^), the intensity of GSB signal in heterojunctions, in Fig. 2d, decreased significantly at 500 fs delay compared to MoS_2_, which may be attributed to electron transfer from MoS_2_ to GaN [54]. The normalized kinetics of A excitons extracted from TA spectra are shown in Fig. 2e. The transient attenuation dynamics can be fitted with tri-exponential function [55], $S_{A} = A_{1} \exp \left( { - \frac{t}{{\tau_{1} }}} \right) + A_{2} \exp \left( { - \frac{t}{{\tau_{2} }}} \right) + A_{3} \exp \left( { - \frac{t}{{\tau_{3} }}} \right)$, where *A*_1_, *A*_2_, and *A*_3_ are the amplitude of lifetime τ_1_, τ_2_, and τ_3_, as shown in the interpolation table. The fast attenuation component τ_1_ can be assigned to the fast capture process of photoexcited carriers [54, 55]. The second component τ_2_ may be related to the exciton–phonon scattering [56]. The slower component τ_3_ may be attributed to radiative charge recombination or relaxation [57]. It can be observed that the much longer recombination lifetime of GaN/MoS_2_/PEDOT:PSS (6.79 ns) compared to that of GaN/MoS_2_ (1.84 ns) and MoS_2_ (0.71 ns), which can be attributed to the enhanced photogenerated carrier separation in the ternary heterojunctions, as reported [58]. The long-lived charge separation state indicates that the hybrid heterostructure has an important application prospect in photovoltaic devices [55–58].

### Photoresponse of GaN NRAs/MoS_2_/PEDOT:PSS Heterostructures

**Fig. 3:**
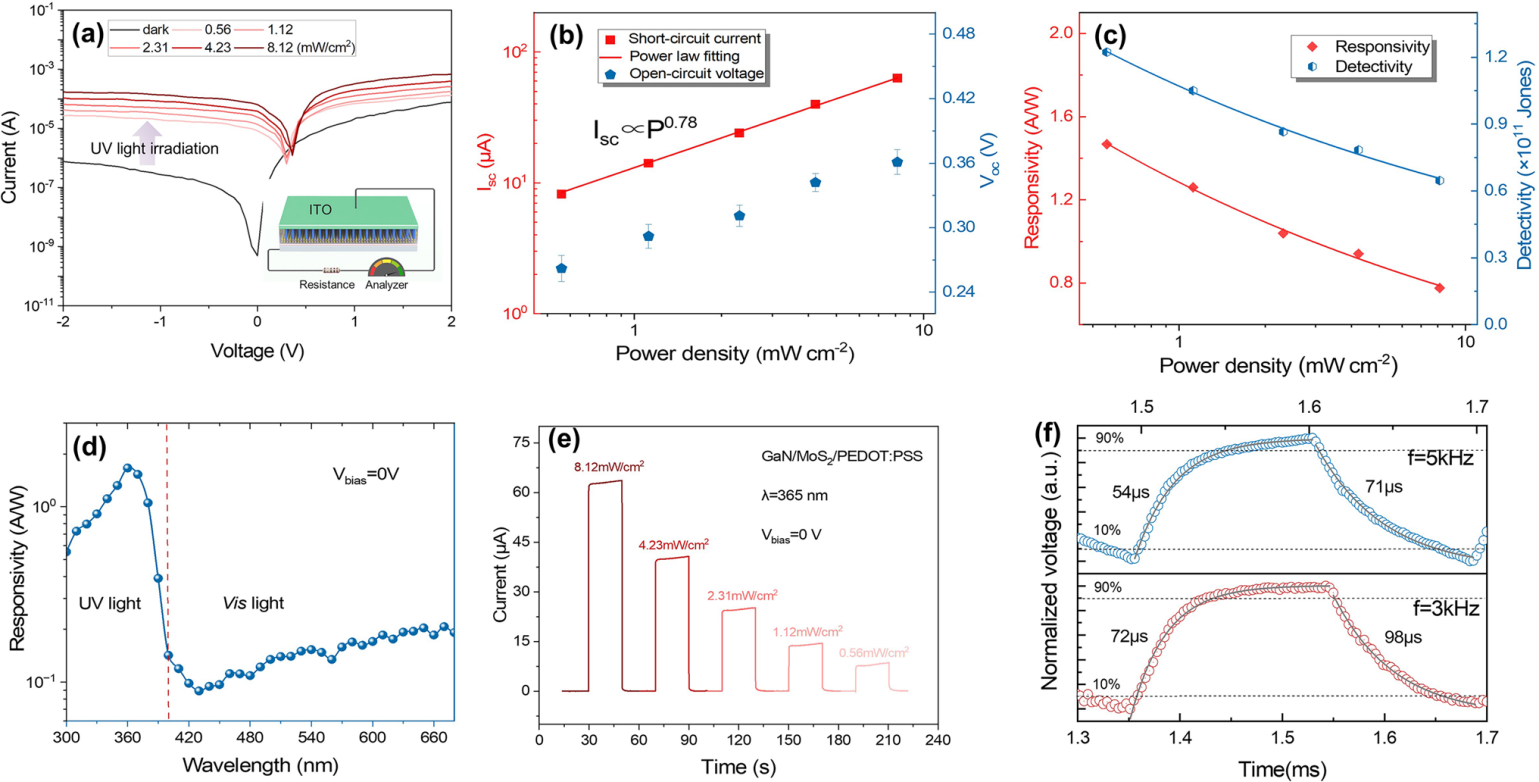
Photoresponse properties of the vertical GaN NRAs/MoS_2_/PEDOT:PSS heterostructure. **a** *I-V* characteristics of the photodetectors under 365 nm UV light with different illumination power density and in the dark. Inset is a schematic diagram of devices. **b** The extracted *I*_sc_ and *V*_oc_ regulation of devices at *V*_ds_ = 0 V.** c** Light power density-dependent responsivity and detectivity. **d** Spectral photoresponse of the responsivity and **e** *I*-*T* curves under 365 nm UV light illumination, as well as **f** time-resolved photoresponse curve of the device under 3 and 5 kHz pulsed UV light in self-powered mode

The photodetection devices based on GaN/MoS_2_/PEDOT:PSS heterojunctions were fabricated, and their photoresponse performances were determined. The schematic structure of GaN/MoS_2_/PEDOT:PSS photodetector is depicted in the illustration of Fig. 3a, where the ITO electrode was deposited on GaN. As shown in Fig. 3a, the typical current–voltage (*I* − *V*) characteristics of the photodetector in logarithmic ordinate scale, with a bias range of − 2 to 2 V, under various intensities of UV light (365 nm) were measured. The photodetector at zero bias exhibits the significant current increases from < 5 × 10^–10^ A in the dark to > 6.3 × 10^–5^ A under light irradiation, demonstrating a remarkable photoelectric sensitivity with light on/off ratio > 1.2 × 10^5^. Besides, the photocurrent of the GaN/MoS_2_/PEDOT:PSS heterojunction is larger than that of GaN/MoS_2_ in Fig. S8, benefiting from the enhanced built-in electric field. As is known, the photogenerated electron–hole pairs would be separated effectively, thus breaking the thermal equilibrium state [59]. Consequently, a short-circuit current (*I*_sc_) and an open-circuit voltage (*V*_oc_) were formed when the device was irradiated by UV light. In Fig. 3b, the dependence of *I*_sc_ and *V*_oc_ on the light power intensity (*P*) was investigated. As *P* increases to 8.12 mW cm^−2^,* I*_sc_ and *V*_oc_ could reach 63.2 µA and 0.36 V, respectively. The nonlinear increasing relationship is fitted by the power law [30] as $I_{{{\text{ph}}}} \propto P_{{{\text{in}}}}^{{\upalpha }}$, where the exponent α indicates the dependence of photocurrent on light intensity. The exponent α is closely related to the generation and trapping of carriers [32]. The fitted value was calculated as 0.78, close to unity, suggesting a few carrier traps in heterojunction. The output electrical power (*P*_el_) describes the electricity generation capability of a photovoltaic device and is defined as *P*_el_ = *I*_ds_*V*_ds_ [60]. As shown in Fig. S9, the *P*_el_ reaches a maximum of 4.34 µW. Besides, the responsivity (*R*) and detectivity (D*) were calculated to quantitatively evaluate the photovoltaic response of device. The responsivity is evaluated with the equation [61] as ${\text{R = }}\frac{{{\text{I}}_{{{\text{ph}}}} {\text{ - I}}_{{{\text{dark}}}} }}{{{\text{P}}_{{{\text{in}}}} {\text{A}}}}$, where *I*_ph_ and *I*_dark_ are the current under illumination and in the dark, *P*_in_ means the power density of the incident light, *A* is the effective area of the device (1 mm^2^). An accurate D*, representing weak light detection capability, can be derived from the noise power spectra, which can be obtained by taking the Fourier transform of dark current traces in Fig. S10. The D* is defined as ${\text{D* = }}\frac{{{\text{R}}A^{1/2} }}{{{\text{S}}_{{\text{n}}} }}$ [62], where ${\text{S}}_{{\text{n}}}$ is the noise spectral density. In Fig. 3c, both* R* and D* monotonically decrease as the light power density increases. The devices under light irradiation with low power density of 0.56 mW cm^−2^ show high *R* of 1.47 A W^−1^ and D* of 1.2 × 10^11^ Jones (1 Jones = 1 cm Hz^1/2^ W^−1^). The external quantum efficiency (EQE) in Fig. S11 was calculated by ${\text{EQE}} = \frac{hc}{{e\lambda }}R$ [30], where parameters *h*, *c,* and λ are the Planck constant, the speed of light, and the wavelength of the incident light. It can be found that the calculated EQE reaches the maximum value of 498%, which indicates a high optical gain. The wavelength-dependent responsivity of the photodetector without bias drive is depicted in Fig. 3d. It can be observed that the peak wavelength of the spectral responsivity curve is located at ~ 365 nm. Furthermore, the device also exhibits visible light detection capability, indicating a promising prospect in multiband photodetection.

Subsequently, the time-resolved photovoltaic response of GaN/MoS_2_/PEDOT:PSS under 365 nm light with different power densities is investigated in Fig. 3e. The photocurrent varies periodically as the light switch, revealing high robustness and good reproducibility. The temporal photoresponse under 5 and 3 kHz pulsed 365 nm light was also evaluated by a digital oscilloscope. Multiple rapid-changing impulse responses were recorded and are displayed in Fig. S12. The response speed was determined by calculating the rise time (*τ*_r_) and fall time (*τ*_f_) from the magnified pulse in Fig. 3f. The photodetector under 5 and 3 kHz pulsed light exhibits ultrafast response to UV light with *τ*_r_/*τ*_d_ = 54/71 µs and *τ*_r_/*τ*_d_ = 72/98 µs, due to its unique advantages of vertical structure.

**Fig. 4:**
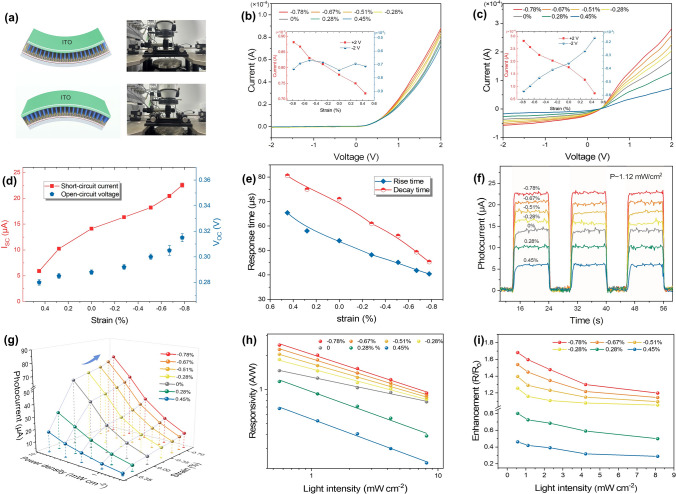
Photodetection performance of the GaN/MoS_2_/PEDOT:PSS heterojunction modulated by the piezo-phototronic effect. **a** Schematic illustration and actual photograph of tensile and compressive strained photodetector. Strain-dependent *I*-*V* characteristics of the device **b** in the dark and **c** under 365 nm illumination with the power density of 1.12 mW cm^−2^. The inset reveals the relationship between output current and strain at the bias of + 2 and − 2 V, respectively. **d** The extracted *I*_sc_, *V*_oc_ and **e** response time, as well as **f** time-dependent photoresponses of the strained device without applying a bias. Variations in **g** photocurrent, **h** responsivity, and **i** relative responsivity changes (*R*/*R*_0_) for different strained photodetectors under increasing illumination power densities

Notably, the strain-modulated photodetection performances of GaN/MoS_2_/PEDOT:PSS have been investigated. The schematic diagram and actual photograph of the 1D/2D device under compressive and tensile strain are shown in Fig. 4a. The *I*-*V* characteristics of photodetectors in the dark were investigated under various strains ranging from − 0.78% to 0.45%, as depicted in Fig. 4b and its inset. In the case of + 2 V forward bias, the current is directly proportional to the compressive strain, whereas it decreases with the increase in tensile strain. Obviously, the output current of 0.78 × 10^–4^ A at the strain-free state can be enhanced to 0.88 × 10^–4^ A under − 0.78% strain. Accordingly, the calculated rectification ratio of heterojunction increased from 105.4 to 120.5, which is associated with the junction barrier modulated by strain-induced piezopotential [25]. Furthermore, the piezo-phototronic effect was demonstrated in the strained device at 365 nm illumination with a power density of 1.12 mW cm^−2^, as shown in Fig. 4c. At + 2 V bias, the highest achieved photocurrent (2.81 × 10^–4^ A) at − 0.78% strain is increased by 58.6% compared to that without strain. As described in Fig. 4d, the effect of strain for *I*_sc_ and *V*_oc_ was extracted from Fig. S13a, which indicates that photovoltaic performance is closely associated with the external strain. The maximum compressive strain of − 0.78% produced a higher photocurrent of 22.5 µA, whereas 0.45% of that exhibited a low photocurrent of 5.9 µA. The self-powered drive capability *P*_el_ tuned by strain was also revealed in Fig. S13b. Furthermore, the time-dependent photoresponse for the strained photodetectors without bias was investigated. The changes in response time are shown in Fig. 4e, which were extracted from the temporal photoresponse in Fig. S14. The response time shows a declining trend with the increase in compressive strain, whereas it slows down with the tensile strain increasing. The photodetectors at − 0.78% strain showed an impressive response time with *τ*_r_ of 40 µs and *τ*_d_ of 45 µs. Consistent responses with on/off switching cycles in Fig. 4f reveal a superior reproducibility for 1D/2D device even in the bent state. It is worth mentioning that the strained photodetectors after repetitive bending operation exhibited promising performance reliability in Fig. S15. After 1000 and 2000 bending cycles at the strain of ~ 1.2%, the retentions of photocurrent at 0 V were 93.7% and 90.6%, respectively.

The photocurrents of the device under various power densities and strains at zero bias were analyzed and are concluded in Fig. 4g. It can be found that the increasing rate of photocurrent is larger for higher power densities, confirming a significant performance improvement by the piezo-phototronic effect [24, 63]. Furthermore, the *R* and D* of photodetector without power drive are further calculated in Figs. 4h and S16, respectively. Both *R* and D* for all power densities gradually increase with increasing compressive strain. Consequently, the 1D/2D device exhibits the highest *R* and D* of 2.47 A W^−1^ and 2.6 × 10^11^ Jones, respectively. The relative responsivity changes are described in Fig. 4i, which is defined as (*R*-*R*_0_)/*R*_0_, where *R* and *R*_0_ represent responsivity with and without adopting applied strains, respectively. The enhancement of responsivity reaches 68.2% at the light irradiation density of 0.56 mW cm^−2^ and strain of − 0.78%. As light power density increases, the enhancement factor gradually decreases, which may be attributed to the saturation and trapping of photogenerated carriers [30].

Benefiting from the combination of 1D/2D array structure and piezo-phototronic effect, the device at compressive strain shows highly outstanding photodetection performance, which, to our best knowledge, are superior to the state-of-the-art self-powered flexible and similar material structured photodetectors, as listed in Table 1, supporting information.

### Detailed Working Mechanism of Strain-Tunable Photoresponse

**Fig. 5:**
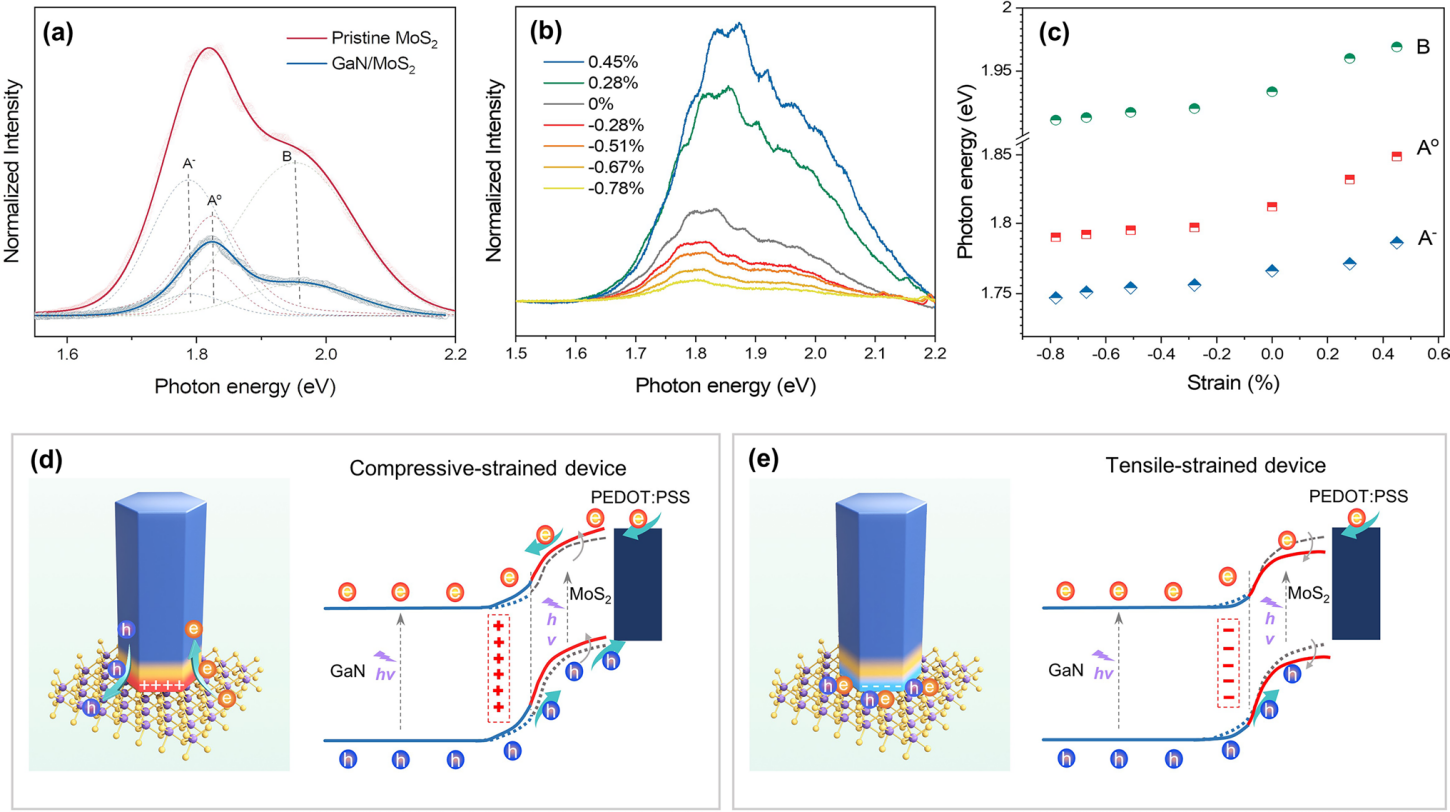
Detailed working mechanism of photoresponse regulated by piezo-phototronic effect. **a** PL spectra of pristine MoS_2_, GaN/MoS_2_ under 532 nm excitation. **b** PL spectra of GaN/MoS_2_ under different strain. **c** Photon energy of A^o^, A^−^ and B as a function of strain. Energy band diagrams of GaN/MoS_2_/PEDOT:PSS heterojunction under UV illumination at **d** compressive strain and **e** tensile strain

The strain effect on interfacial carrier behaviors of GaN/MoS_2_ heterojunction was profoundly studied by PL spectrum under 532 nm laser excitation. In a strain-free state, the PL intensity of GaN/MoS_2_ was suppressed relative to pristine MoS_2_ in Fig. 5a, indicating a type-II band alignment of heterojunction for efficient charge transfer [64]. For the spin–orbit coupling of valence band, each PL spectra can be deconvoluted by the B exciton, neutral exciton (A^o^), and trion component (A^−^), using Lorentzian fitting functions [65]. The main PL emission of the pristine MoS_2_ is dominated by trion, whereas that in the heterostructure is A^o^ because trion excitons are almost depleted in charge transfer process [66, 67]. The physical phenomenon was also confirmed by power-dependent (0.008–5 mW) PL spectra in Fig. S17. The PL spectra of GaN/MoS_2_ heterojunction under different strains are shown in Fig. 5b, which reveals a significant enhancement of peak intensity from compressive strain to tensile strain. Each spectrum normalized by peak area is shown in Fig. S18. Obviously, the extracted excition peaks including A^o^, A^−^, and B, in Fig. 5c, showed an obvious blueshift under tensile strain. It has been reported that MoS_2_ PL peak appears a redshift under tensile strain, only taking account of the effect of strain-induced lattice deformation on electronic structure [66, 68], which is contrary to our experimental results. It means that the variation of MoS_2_ can be ascribed to piezopotential, which is similar to that of electrostatic gating [69]. As a result, the separation of photogenerated charge in MoS_2_ was promoted by piezopolarization charge under compressive strain, resulting in significant suppression of PL intensity. In contrast, the PL intensity increased significantly for tensile sample due to the enhanced photoexciton recombination.

In order to further explore the strain-gating mechanism for the self-integrated device, the band structure diagram of GaN/MoS_2_/PEDOT:PSS heterojunction under compressive and tensile strain is plotted in Fig. 5d, e. Considering the atomically thin MoS_2_ film with three layers, MoS_2_ would be fully depleted after contact with GaN. As is known, GaN NRAs with non-centrosymmetric wurtzite structure exhibit piezoelectric effect under strain stimulation [63, 70]. The simulations of the GaN nanowire piezopotential distribution for different strains are shown in Fig. S19. The positive piezopolarization charges generated in GaN NRAs at compressive strain state attract the accumulation of negative charges on the side of MoS_2_, which further increases band slope for MoS_2_. Therefore, the separation and transport of photogenerated carriers are more efficient with additional driving forces of positive piezopotential, thereby improving the photoresponse of GaN/MoS_2_/PEDOT:PSS photodetector in self-powered mode. On the other hand, the negative polarization charges are produced in GaN when the heterojunction suffers from tensile strain. Accordingly, the band slope of MoS_2_ decreases under negative piezopotential, which suppresses the photogenerated carrier transport, thus deteriorating the photoresponse of overall. In conclusion, the above experimental and theoretical analyses reveal that strain is capable of function as another freedom degree to adjust the photogenerated carrier behavior of 1D/2D, which provides a promising strategy for improving the performance of optoelectronic.

### Practical Application of GaN NRAs/MoS_2_/PEDOT:PSS Heterojunctions

**Fig. 6:**
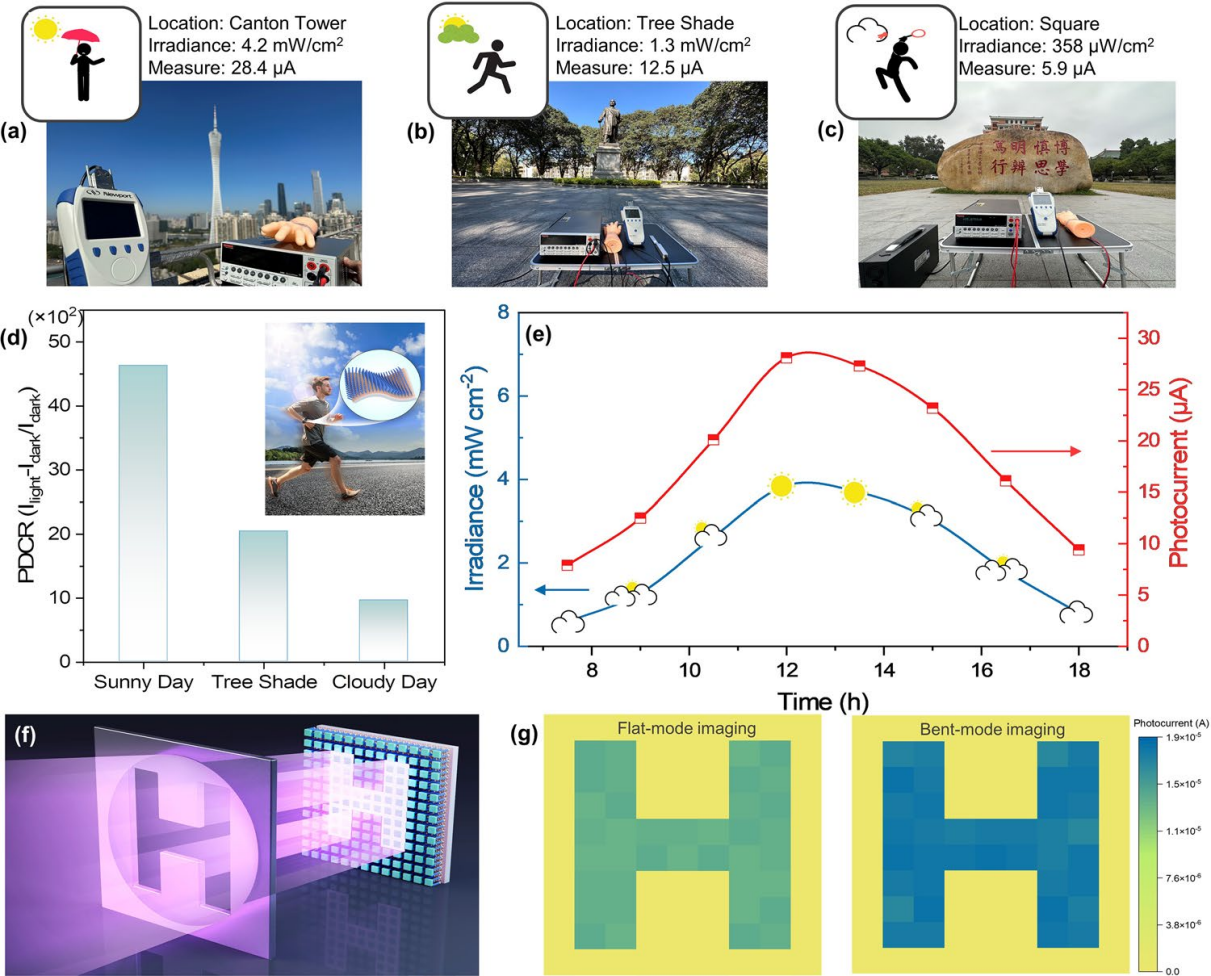
GaN/MoS_2_/PEDOT:PSS for real-time UV monitoring and imaging output. The self-integrated devices attached to a hand, measuring the UV irradiance of **a** Canton tower (sunny), **b** tree shade (sunny) and **c** square (cloudy). **d** The corresponding photo-to-dark current ratios. **e** Real-time photocurrent recorded by the photodetectors throughout the day, varying with the UV intensity. **f** The schematic of the image sensing analysis system. **g** Clear imaging output “H” from the sensor arrays at flat and − 0.51% strained states, at 365 nm illumination of 1.12 mW cm^−2^

To demonstrate the feasibility of 1D/2D photodetector applied in flexible wearable components, the UV photodetection of device attached to a human hand was investigated under different environments, including sunny, tree shade and cloudy conditions. The source meter connected to the self-powered device was used to record real-time dynamic variation in the photocurrent, and the irradiance of 365 nm light from the surroundings was measured by the power meter. As described in Fig. 6a–c, the radiation power densities in sunny, cloudy, and shade environments were 4.2, 1.3, and 0.35 mW cm^−2^ and the photocurrent was detected as 28.4, 12.5, and 5.9 μA, respectively. The corresponding photo-to-dark current ratios (PDCR) [71] in Fig. 6d, defined as (*I*_light_-*I*_dark_)/*I*_dark_, were 4.6 × 10^3^, 2.1 × 10^3^, and 0.96 × 10^3^, which confirms that the as-prepared photodetector can sensitively distinguish different levels of UV light. In Fig. 6e, the real-time photocurrent of the detector was recorded throughout the day, which varies with the UV intensity. Consequently, the integrated device is capable of alerting to excessive UV exposure, which is of great significance in the prevention of skin cancer [72].

Furthermore, a device array based on GaN/MoS_2_/PEDOT:PSS has been demonstrated in imaging sensors. The schematic of the image sensing analysis system is described in Figs. 6f and S20 with a light source, metal mask, flexible sensor array, measurement unit (SMU), and computer. All imaging tests were performed at 0 V bias. As the incident light passes through the letters on the metal mask, the photocurrent generated by the corresponding pixels in the detector array (10 × 10) is received by the SMU. The computer records the size of the current value from SMU and draws pictures in turn. Benefiting from the good uniformity of photocurrents, the sensor arrays in both flat and curved states show the clear imaging output “H” in Fig. 6g, proving its application in flexible high-resolution imaging.

## Conclusion

In summary, we have fabricated wafer-scale GaN NRAs/MoS_2_/PEDOT:PSS heterojunctions for self-powered flexible photodetectors through vdW epitaxy and PMMA-assisted wet transfer. The hybrid device at 365 nm UV illumination exhibits a remarkable self-powered photodetection capability of an ultrahigh *R* of 1.47 A W^−1^, a D* of 1.2 × 10^11^ Jones, and a fast rise/fall time of 54/71 µs, as well as excellent photoresponse repeatability and stability. The striking photovoltaic response effect can be attributed to two aspects. On the one hand, the vertical 1D/2D heterostructure integrated into a photodetector has the following superiority: (i) The strong light absorption of GaN NRAs with huge surface-to-volume ratio could result in the enhanced photogenerated current density. (ii) The 1D/2D quantum confinement for the depletion layer reduces the transit time of photogenerated carriers. On the other hand, the type-II band alignment of GaN/MoS_2_/PEDOT:PSS facilitates the efficient separation of photogenerated carriers. Furthermore, the strain-tunable performance of photodetector was demonstrated by utilizing the piezo-phototronic effect. Notably, the responsivity at compressive strain of − 0.78% reaches up to 2.47 A W^−1^, which is increased by 68.2% compared to the unstrained state. From the experimental and theoretical analysis, the enhanced photoresponse can be attributed to the energy band tilt at the GaN/MoS_2_ interface caused by compressive strain-induced piezopolarization charges promoting the photogenerated carrier separation and transport. In addition, the integrated photodetectors can be integrated into the flexible wearable components for UV sensing and high-resolution imaging. The tunable 1D/2D devices offer a new platform for the development of self-powered, flexible optoelectronics.

## Supplementary Information

Below is the link to the electronic supplementary material.

Supplementary file1 (DOCX 4795 KB)
