# Supplementary material for: Wafer-Scale Vertical 1D GaN Nanorods/2D MoS2/PEDOT:PSS for Piezophototronic Effect-Enhanced Self-Powered Flexible Photodetectors
Source: Nanomicro Lett. 2024 Nov 5;17:56. doi: 10.1007/s40820-024-01553-8 (PMC11534966; doi:10.1007/s40820-024-01553-8)
Supplement: Supplementary file 1 — Supplementary file1 (DOCX 4795 KB) [file 40820_2024_1553_MOESM1_ESM.docx]

Supporting Information for

Wafer-Scale Vertical 1D GaN Nanorods/2D MoS_2_/PEDOT:PSS for Piezophototronic Effect Enhanced Self-Powered Flexible Photodetectors

Xin Tang^1,2^, Hongsheng Jiang^1,2^, Zhengliang Lin^1,2^, Xuan Wang^1,2^, Wenliang Wang^1,2,^* and Guoqiang Li^1,2,^*

^1^ State Key Laboratory of Luminescent Materials and Devices, South China University of Technology, Guangzhou 510640, P. R. China

^2^ Department of Electronic Materials, School of Materials Science and Engineering, South China University of Technology, Guangzhou 510640, P. R. China

*Corresponding authors. E-mail: [wenliangwang@scut.edu.cn](mailto:wenliangwang@scut.edu.cn) (Wenliang Wang); [msgli@scut.edu.cn](mailto:msgli@scut.edu.cn) (Guoqiang Li)

**Supplementary Figures**


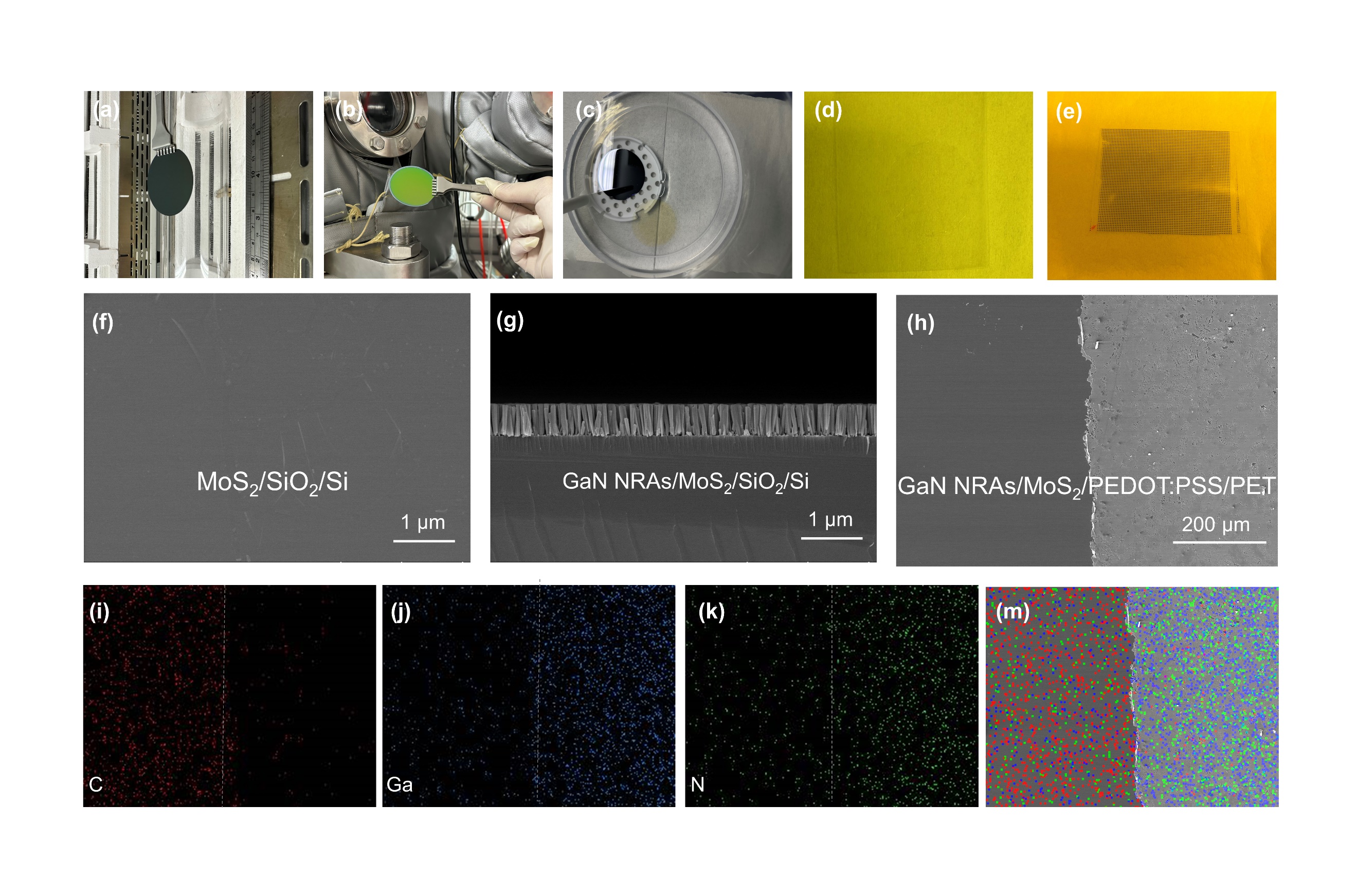


**Fig. S1 a-c** Fabrication process of vertical heterostructures for flexible photodetector arrays. (**a)** 2-inch MoS_2_/SiO_2_/Si prepared by CVD, **(b)** GaN NRAs/MoS_2_/SiO_2_/Si prepared by MBE, **(c)** GaN NRAs/MoS_2_ etched by HF, **(d)** GaN NRAs/MoS_2_/ PEDOT:PSS flexible heterojunction, **(e)** GaN NRAs/MoS_2_/PEDOT:PSS flexible photodetector arrays, and corresponding **(f-h)** SEM images. **i-m** EDS mapping of GaN NRAs/MoS_2_/PEDOT:PSS heterojunction.


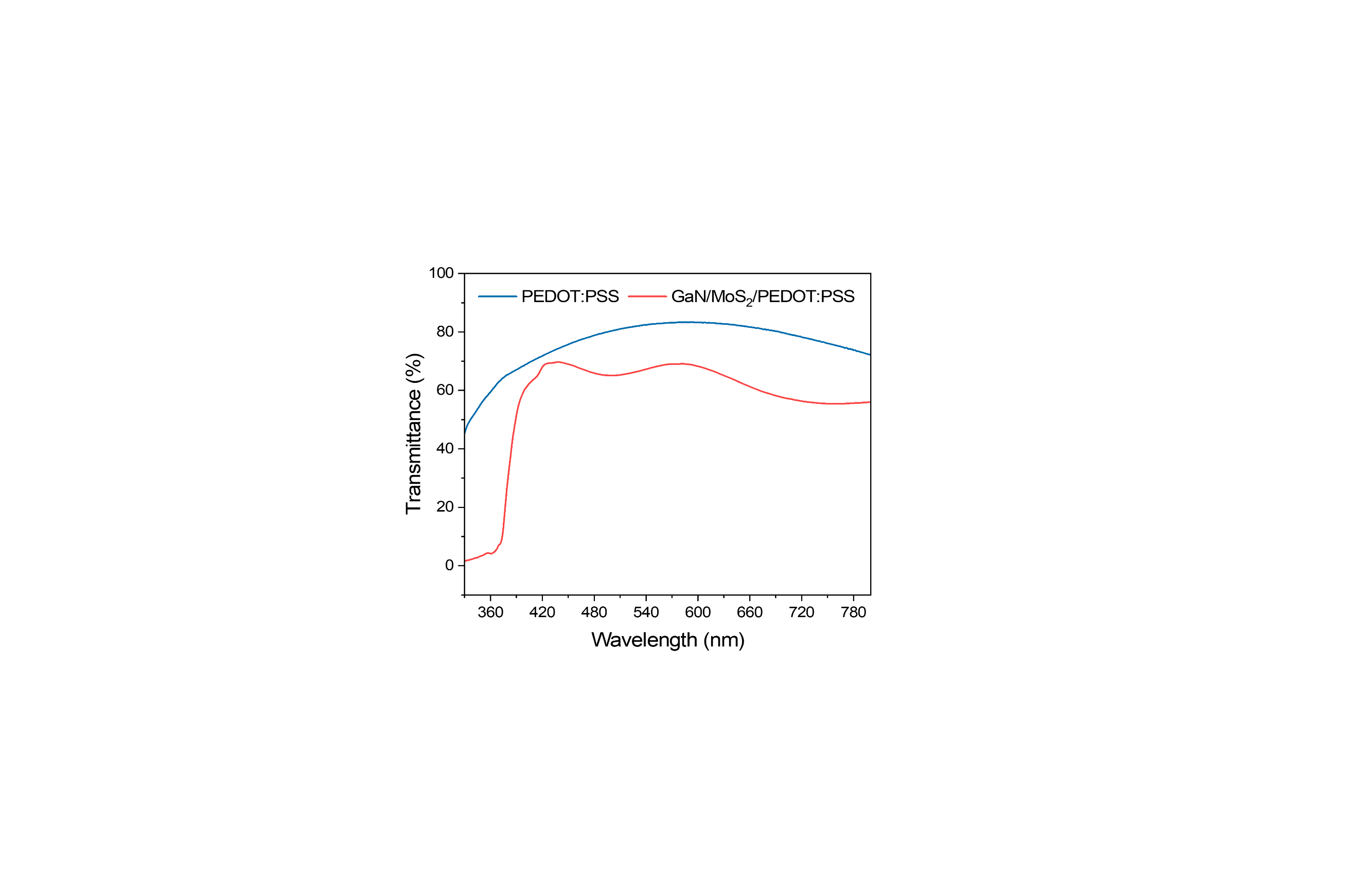


**Fig. S2** Transmittance before and after GaN NRAs/MoS_2_ was transfer onto PEDOT:PSS/ITO/PET


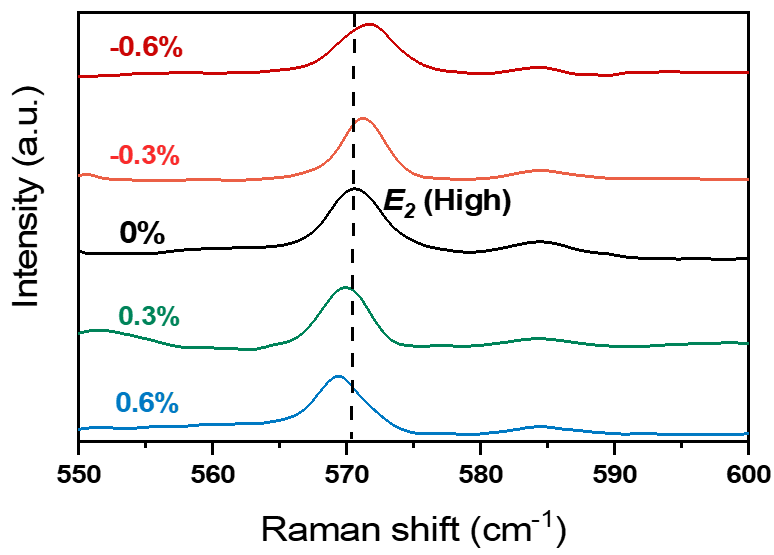


**Fig. S3** Raman spectra of vertical GaN/MoS_2_/PEDOT:PSS heterojunction at different strains


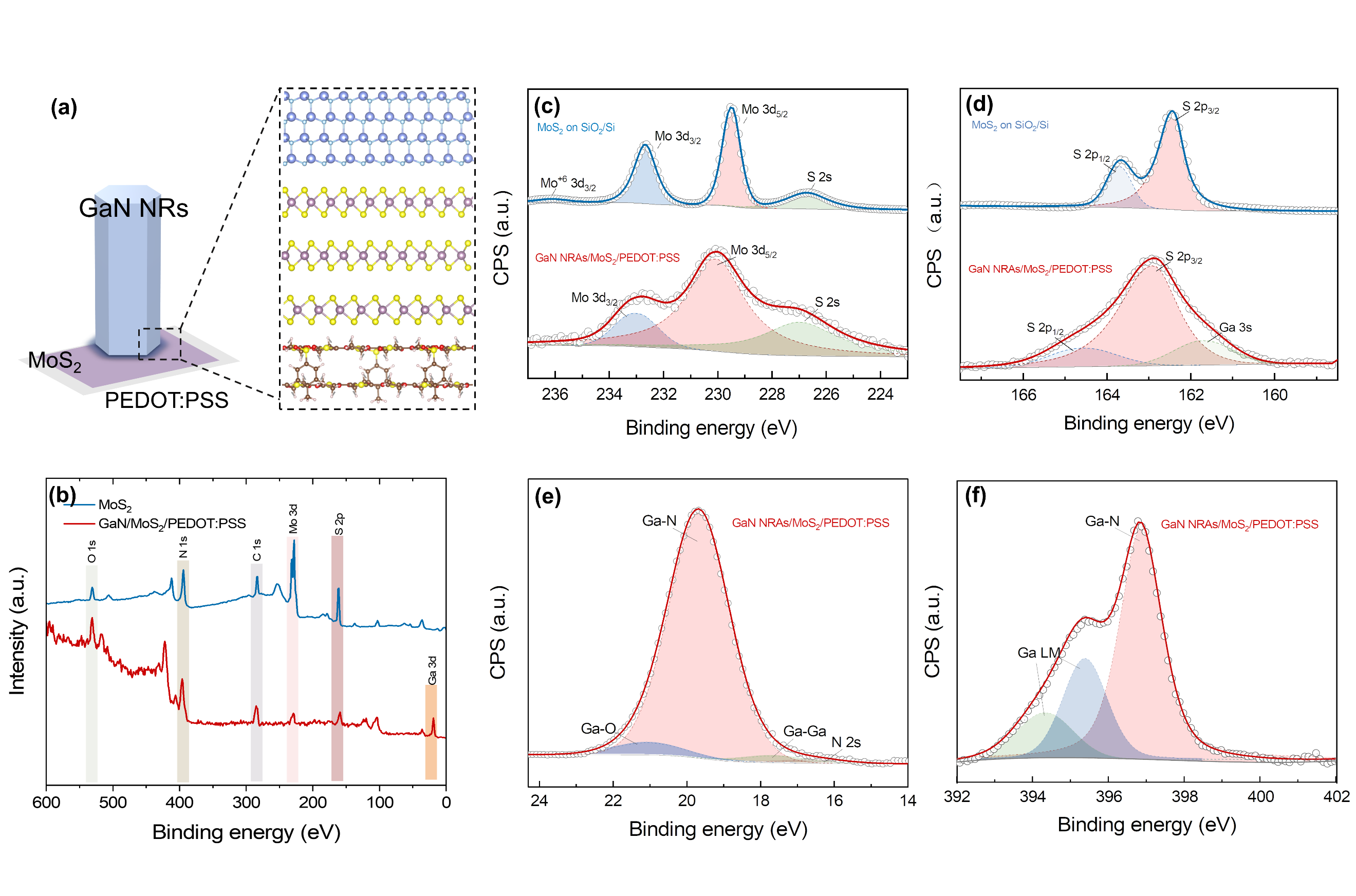


**Fig. S4** **a** Schematic diagram and simulated atomic configuration of the vertical heterostructure. **(b)** XPS full survey spectra of GaN/MoS_2_/PEDOT:PSS and original MoS_2_. **(c-f)** Mo 3d, S 2p, Ga 3d and N 1s XPS spectra for GaN/MoS_2_/PEDOT:PSS, refered to fitted XPS spectra for pure MoS_2_/SiO_2_/Si


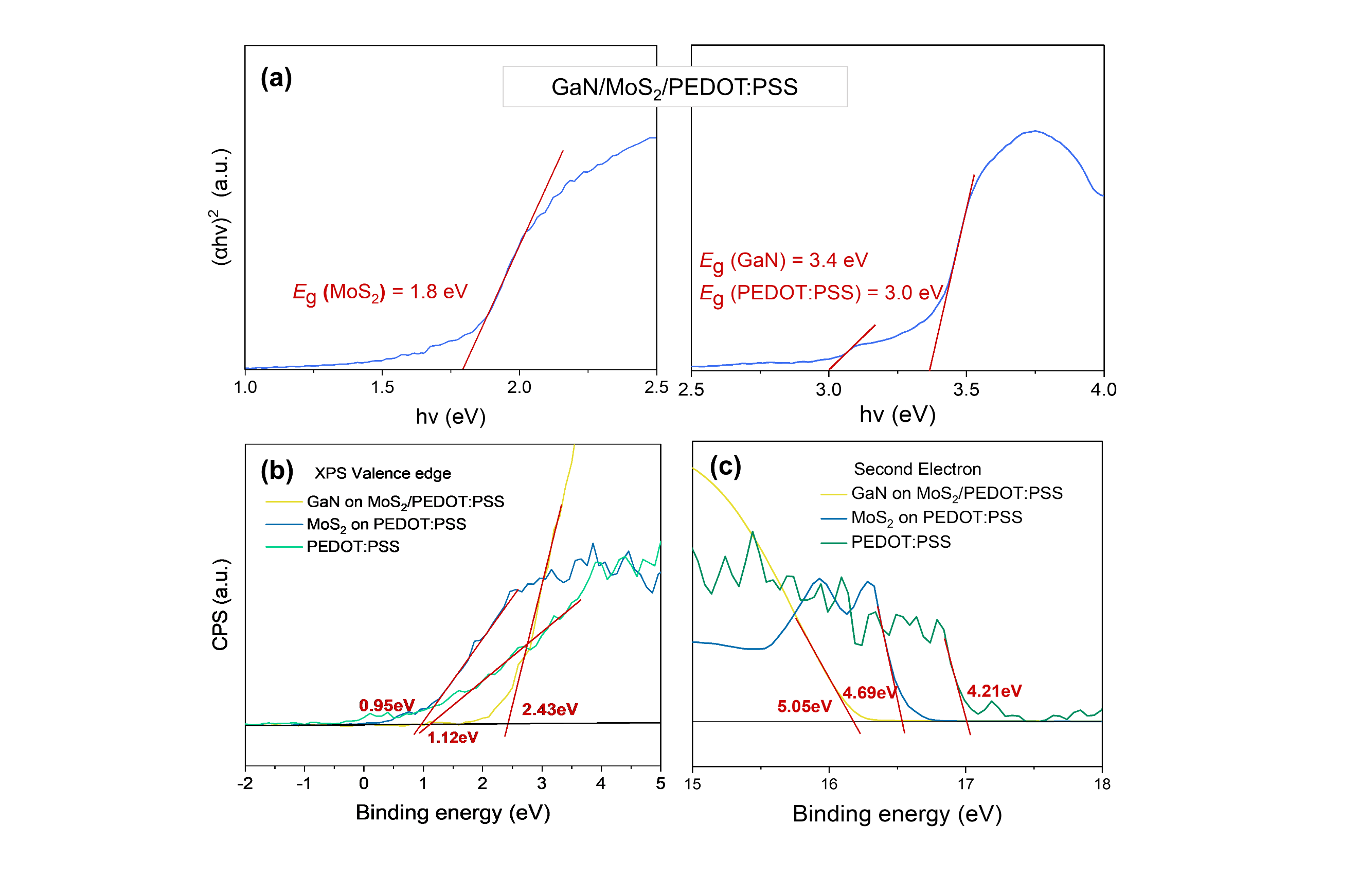


**Fig. S5 a** UV-vis absorption spectrum for GaN/MoS_2_/PEDOT:PSS. **(b)** XPS valence band edge (VB) and **(c)** ultraviolet photoelectron spectra (UPS) work function spectra for MoS_2_, PEDOT:PSS and GaN NRAs. The work functions (Φ) can be obtained from the difference between the photon energy of radiation (21.21 eV) and the second electron cutoff energy


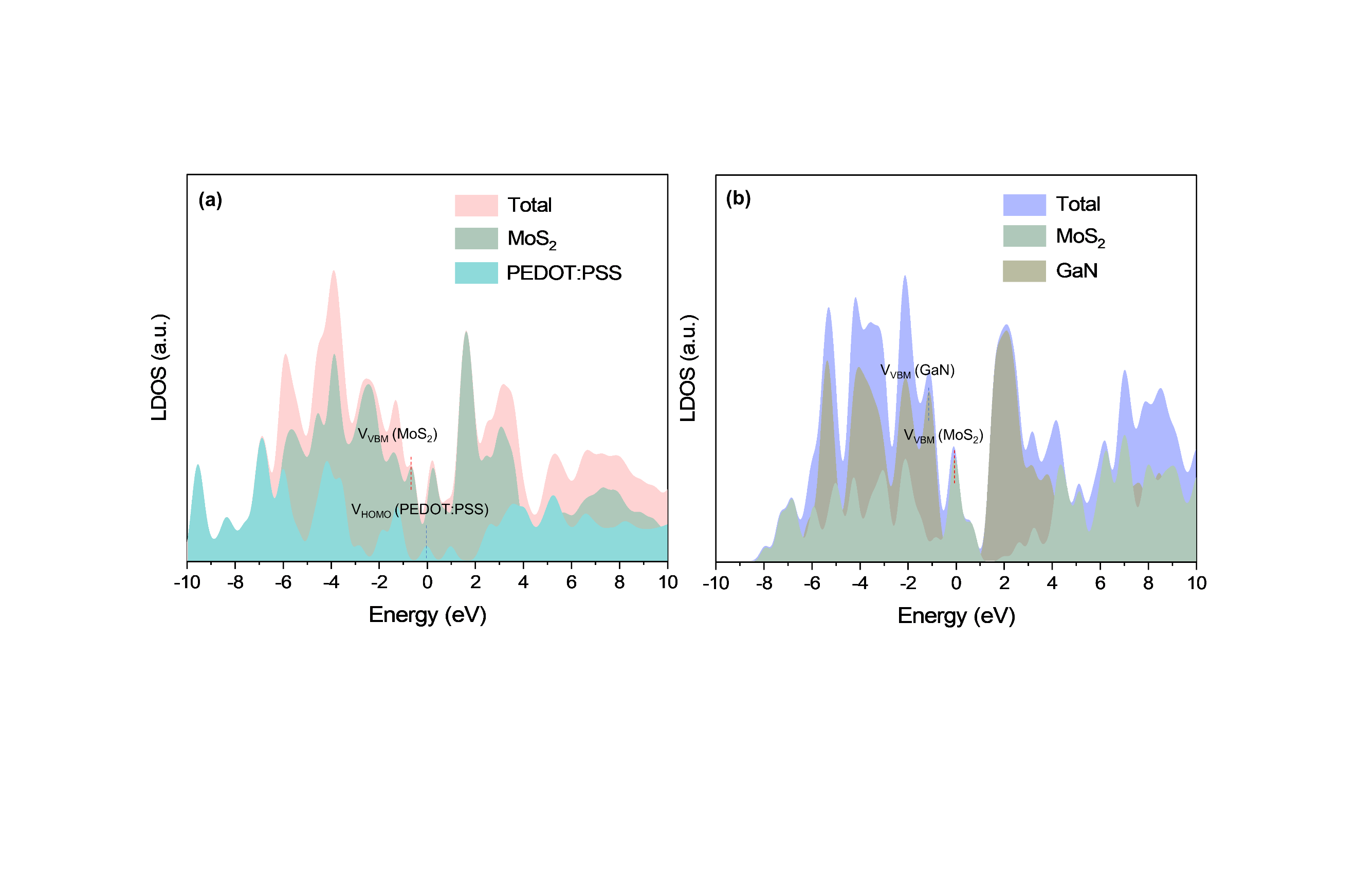


**Fig. S6** The local density of states (LDOS) of hybrid heterojunction


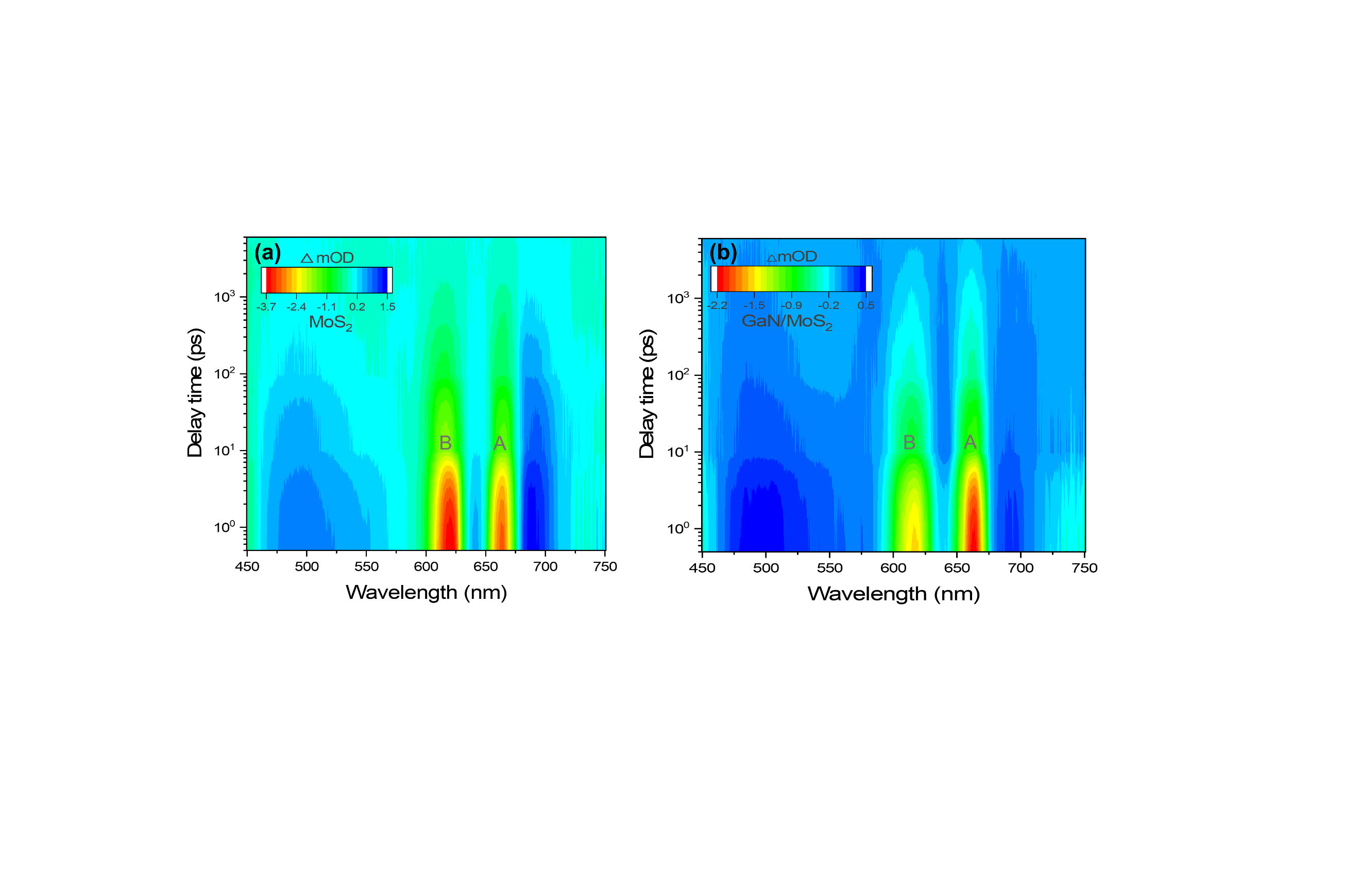


**Fig. S7** Transient absorption (TA) mapping of MoS_2_ and GaN/MoS_2_ at different delays


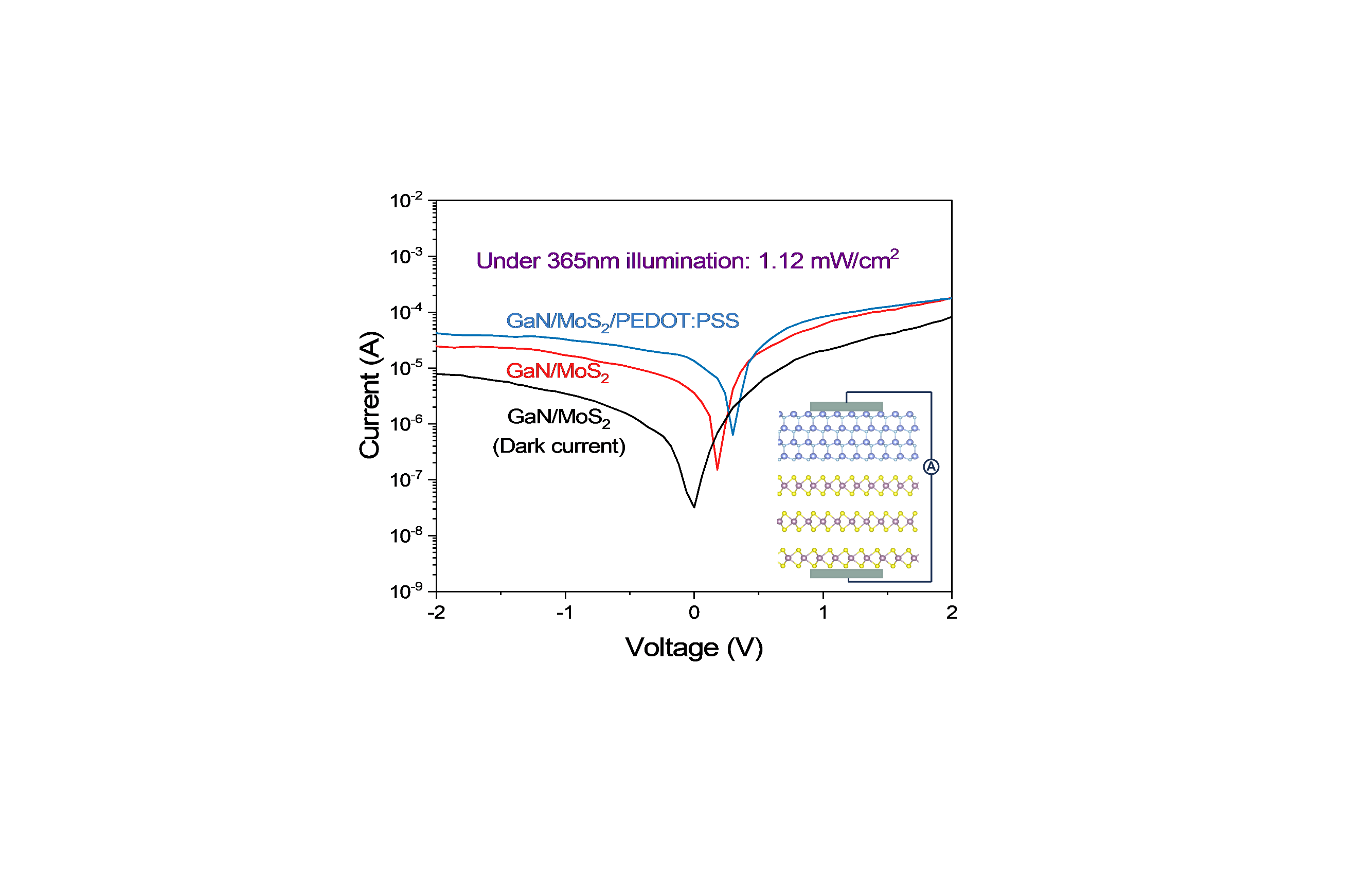


**Fig. S8** Typical I–V characteristics (in logarithmic ordinate scale) of GaN/MoS_2_ and GaN/MoS_2_/PEDOT:PSS photodetectors under 365 nm illumination of 1.12 mW/cm^2^


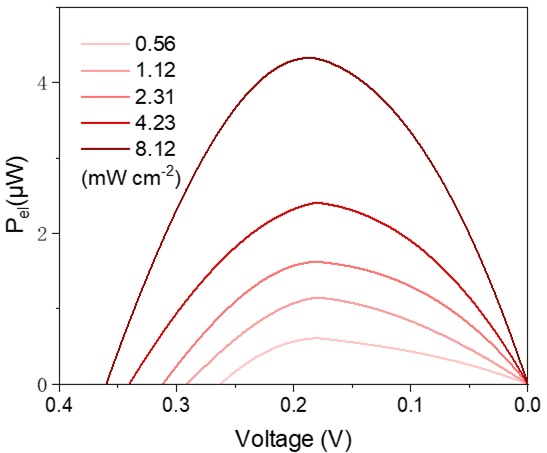


**Fig. S9 a** The output electrical power (*P*_el_) of GaN/MoS_2_/PEDOT:PSS heterojunction.


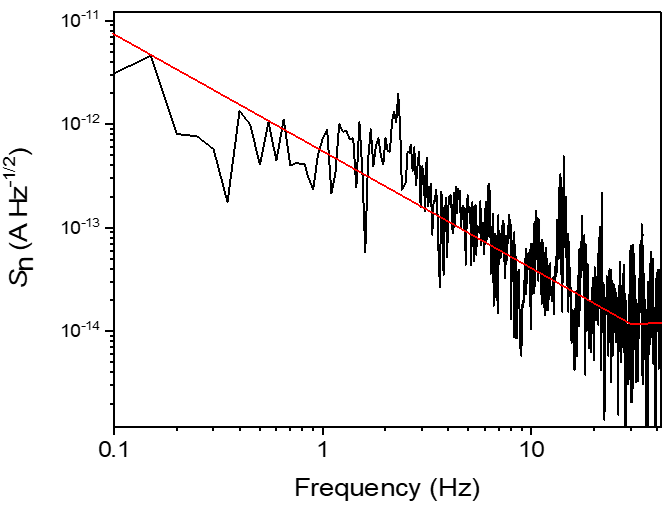


**Fig. S10** The noise spectral densities (*S*_n_) as a function of frequency by taking the Fourier transform of dark current traces


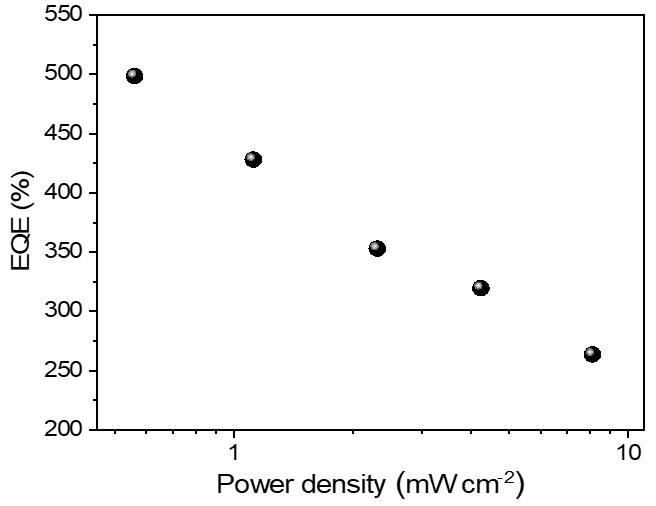


**Fig. S11** The EQE as function of light power density


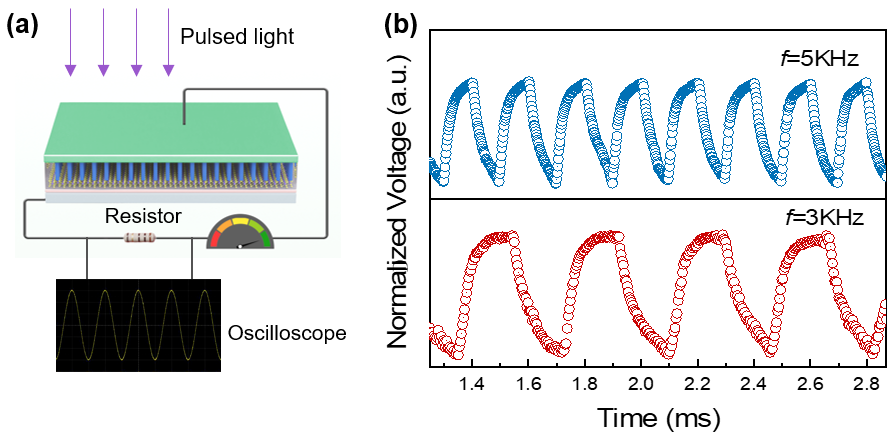


**Fig. S12** (**a**) The schematic of the response time test and (**b**) multiple rapid-changing impulse responses under 5kHz and 3 kHz pulsed 365 nm light


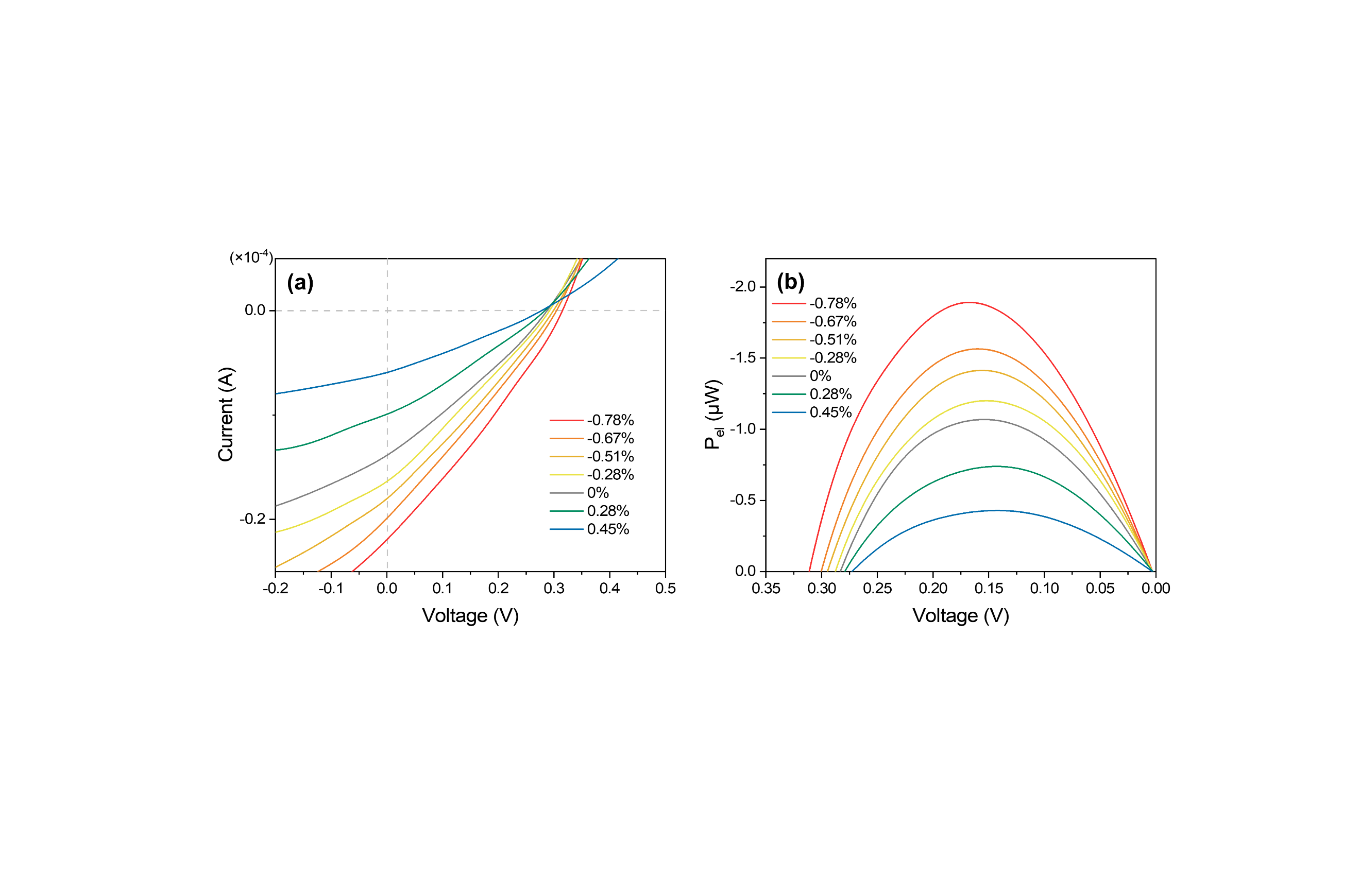


**Fig. S13** (**a**) I-V characteristics and (**b**) *P*_el_ of the photodetectors at different strain


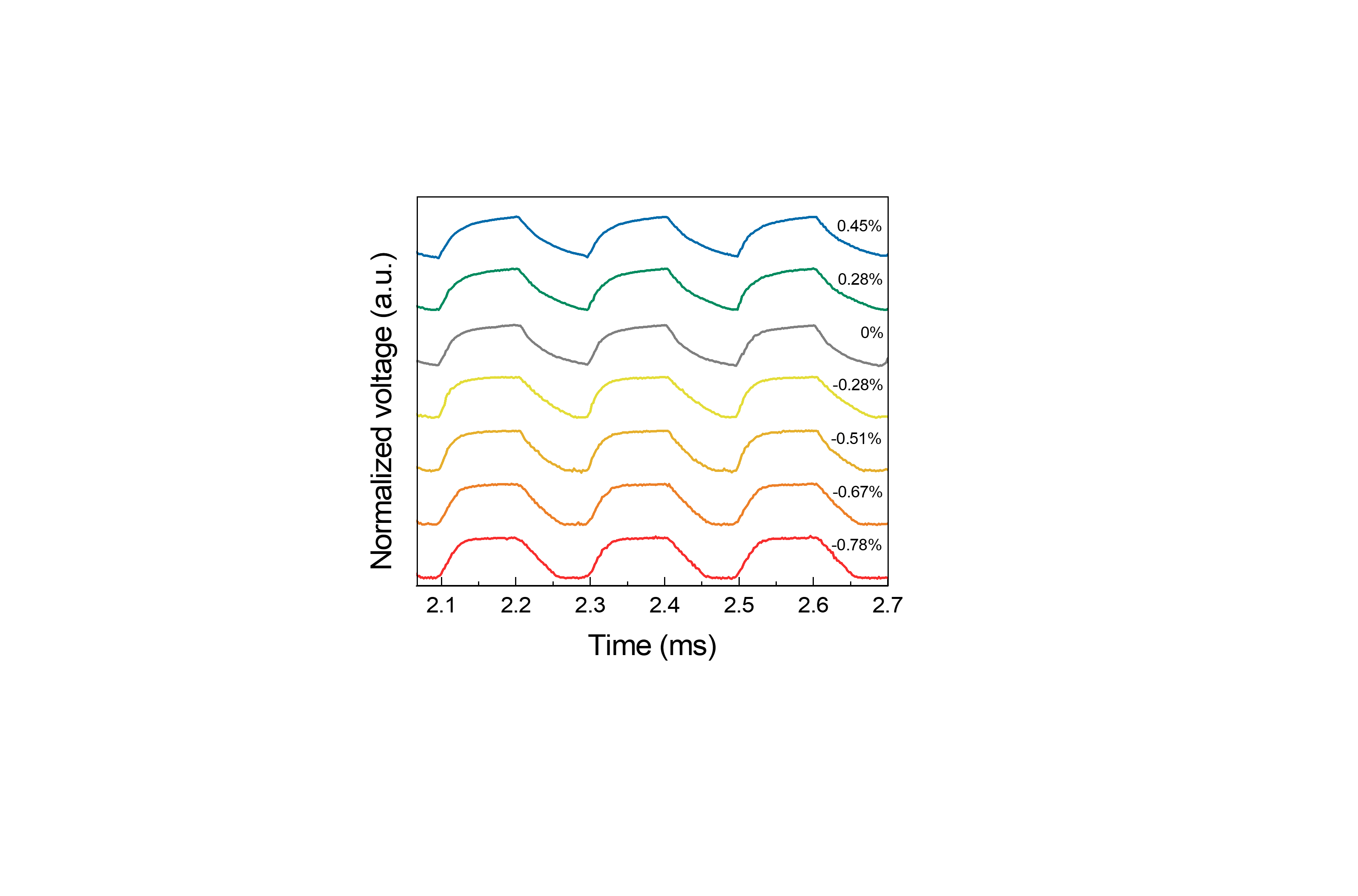


**Fig. S14** The temporal photoresponse of device at different strain


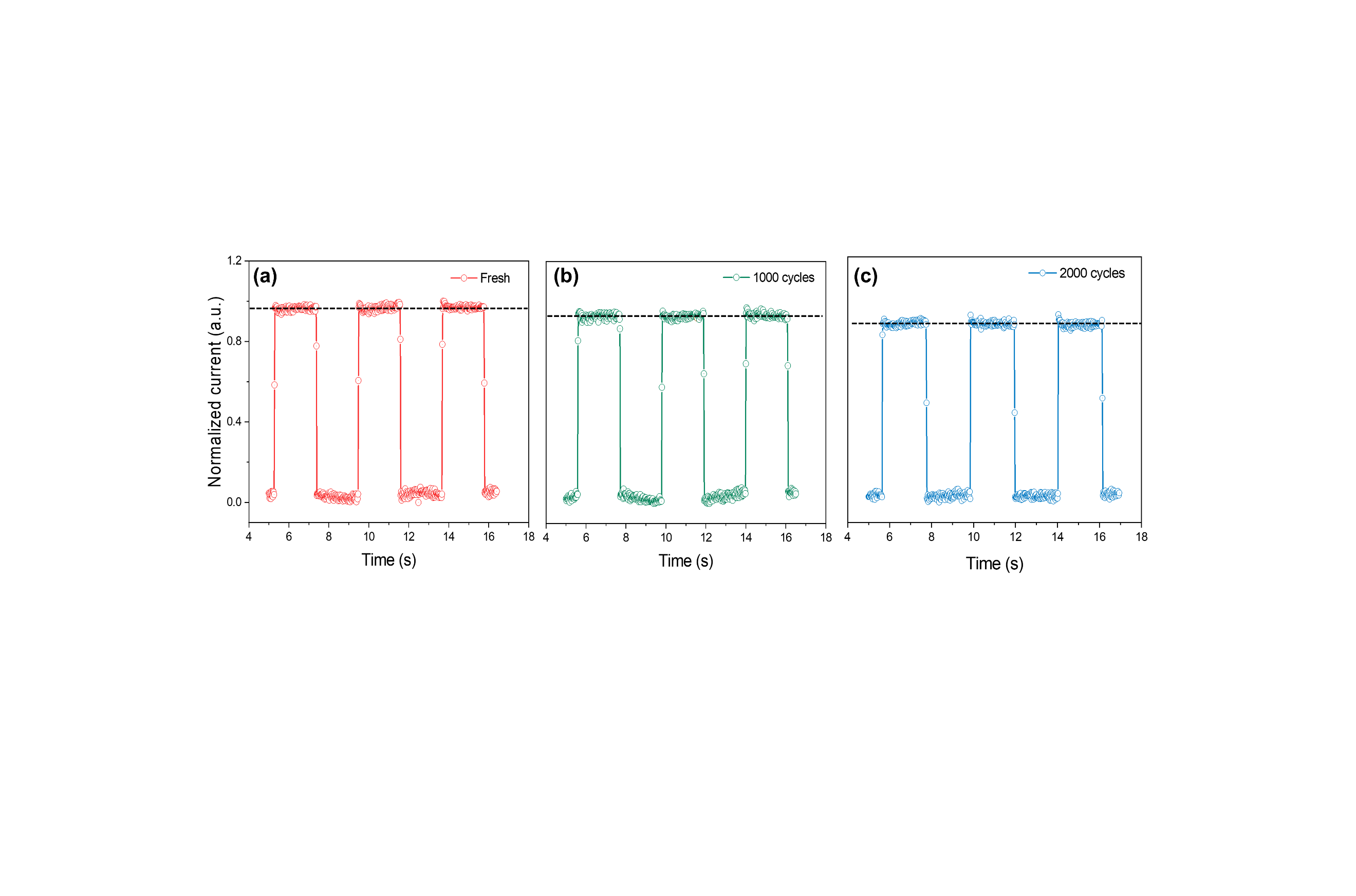


**Fig. S15** The photoresponse of device (**a**) at fresh state and after (**b**) 1000 and (**c**) 2000 bending cycles


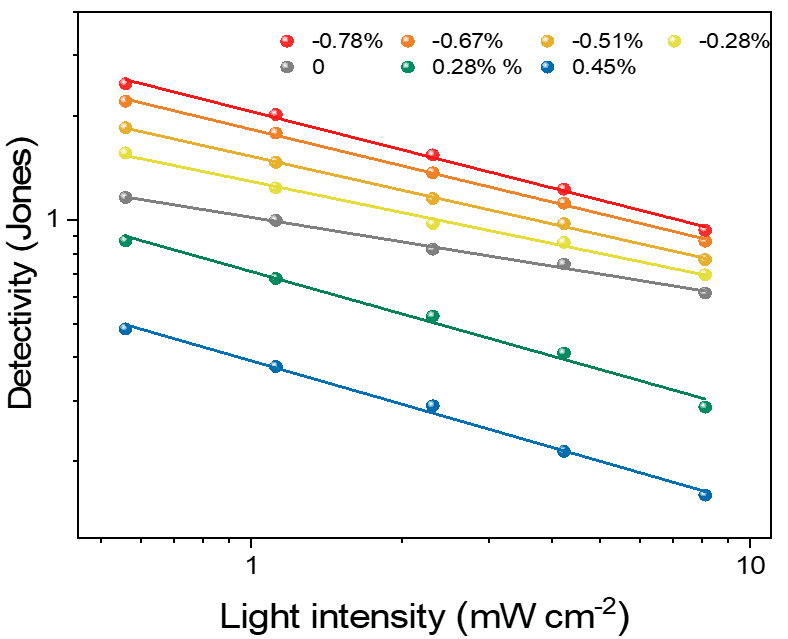


**Fig. S16** Variations in detectivity for the different strained photodetectors under increasing illumination power densities


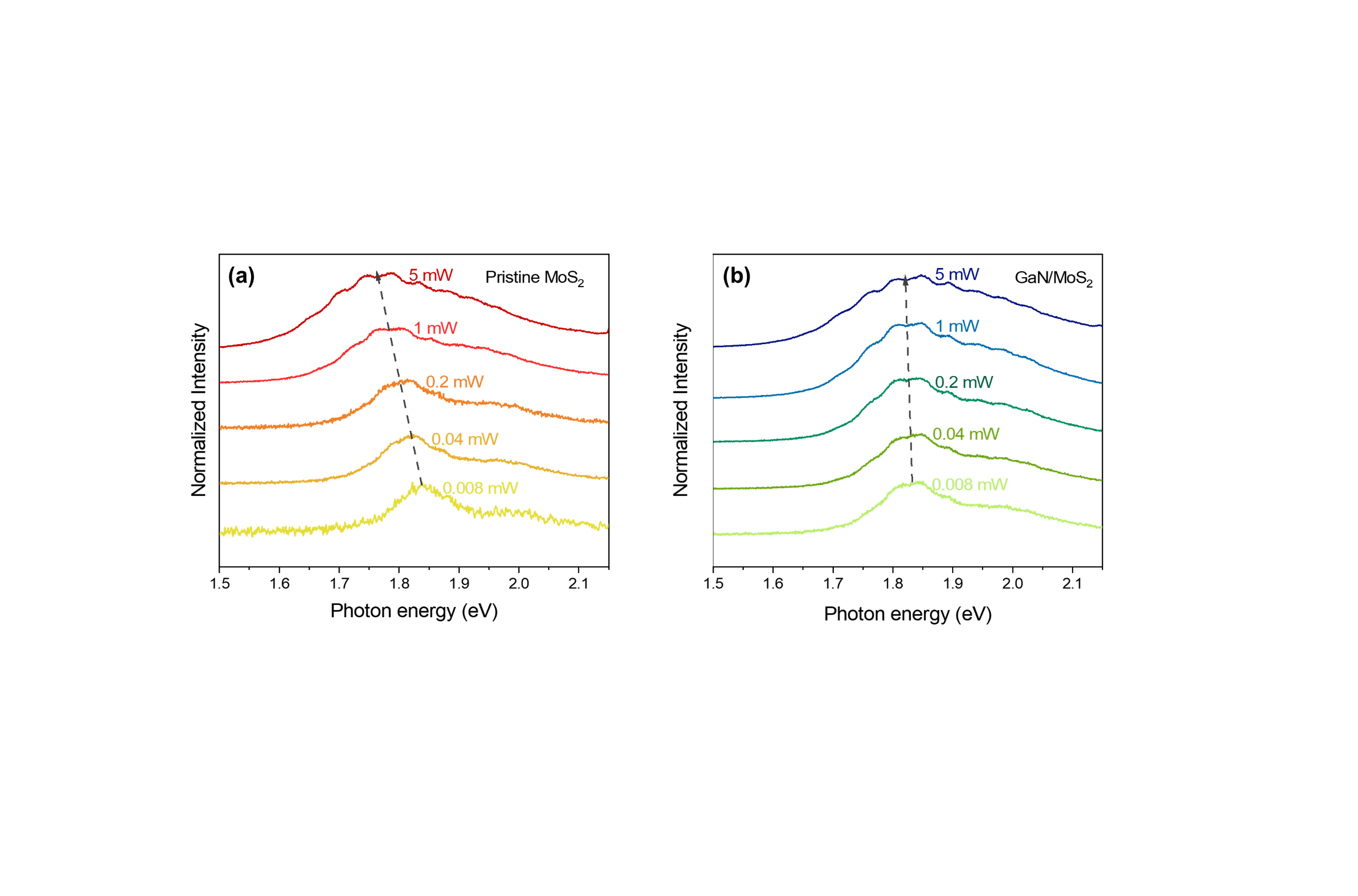


**Fig. S17** Power-dependent (0.008~5 mW) PL spectra for MoS_2_ and GaN/MoS_2_


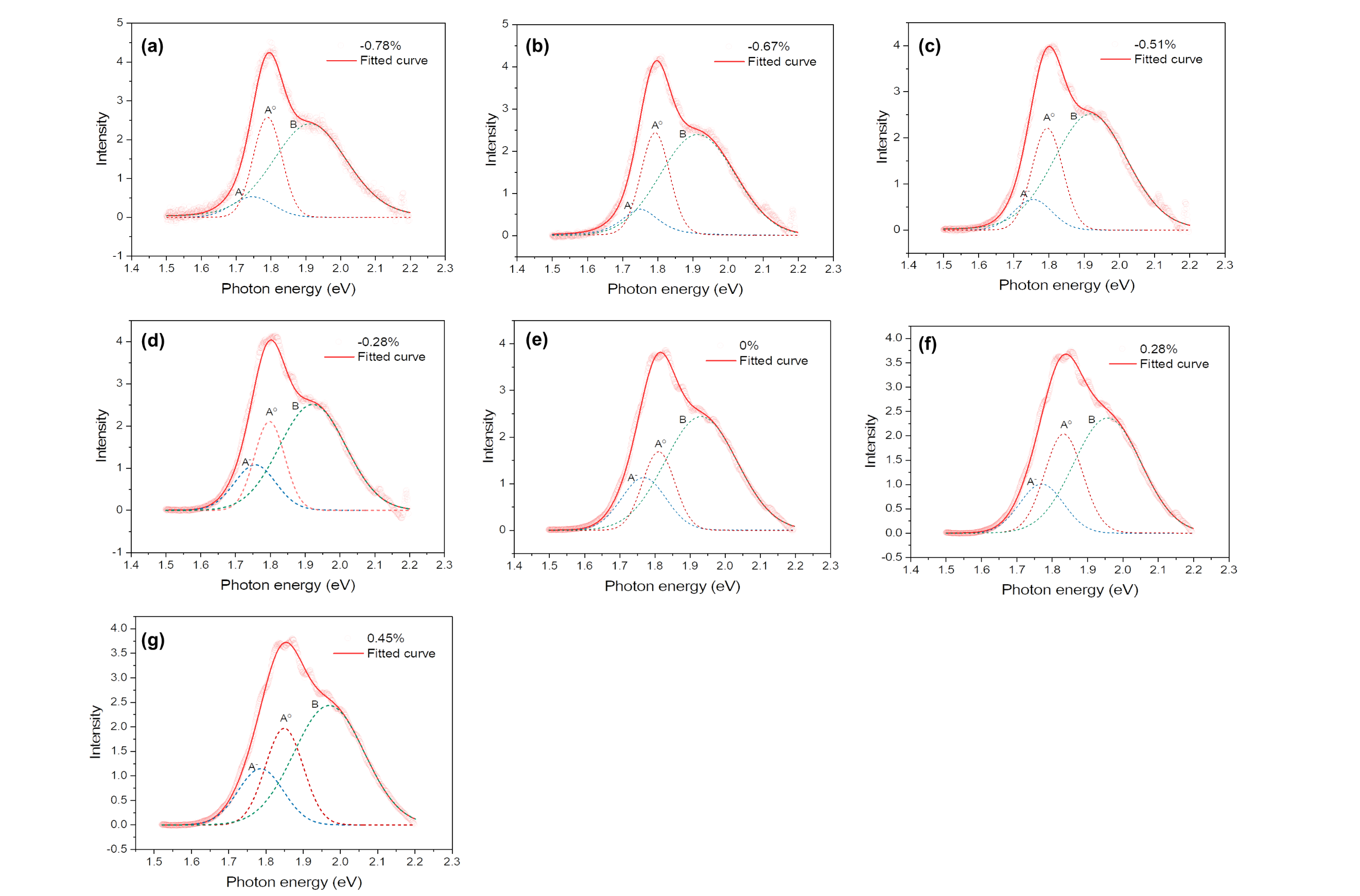


**Fig. S18** PL spectrum at different strains normalized by peak area


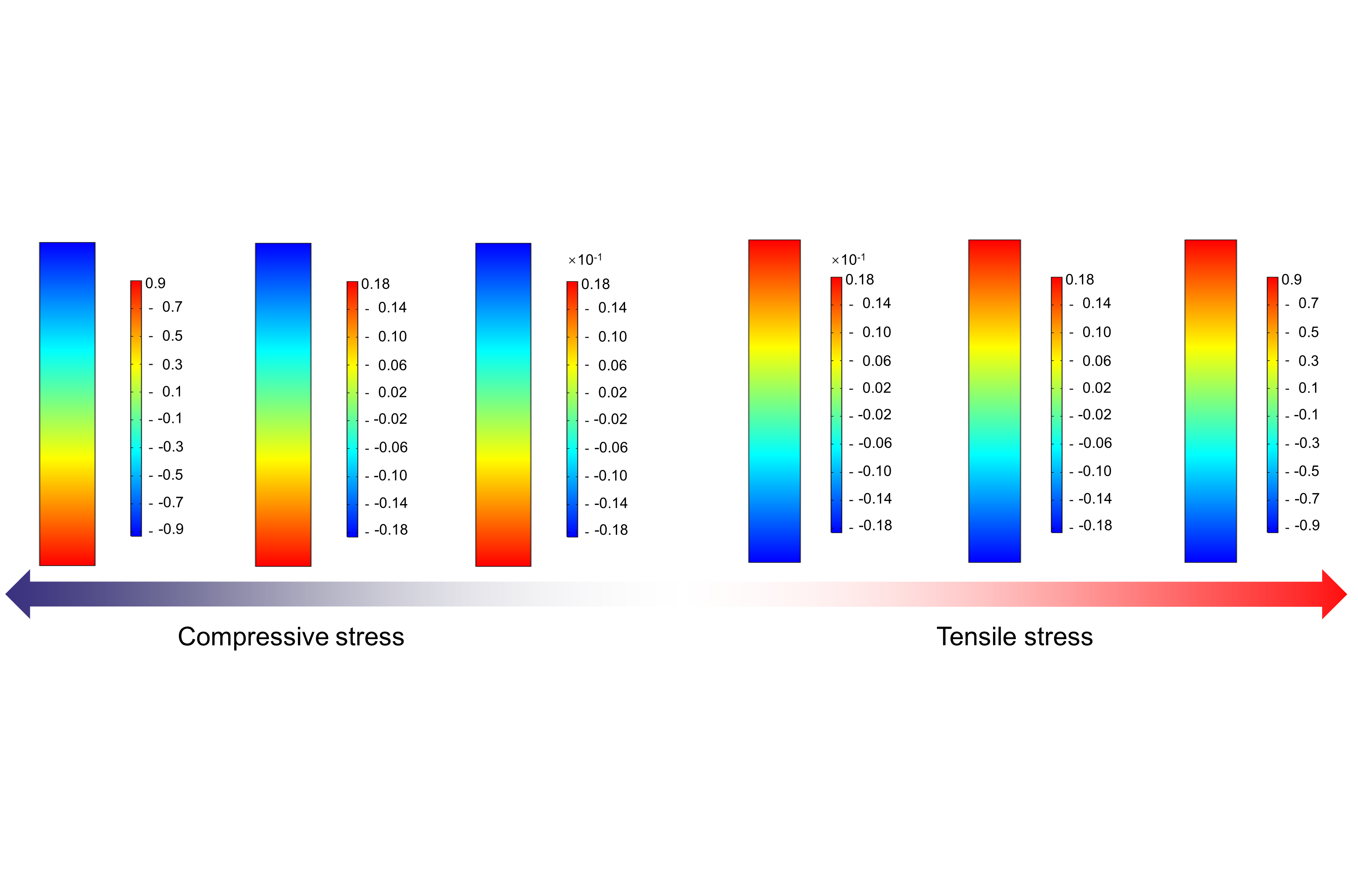


**Fig. S19** The simulations of the GaN nanowire piezopotential distribution for different strains


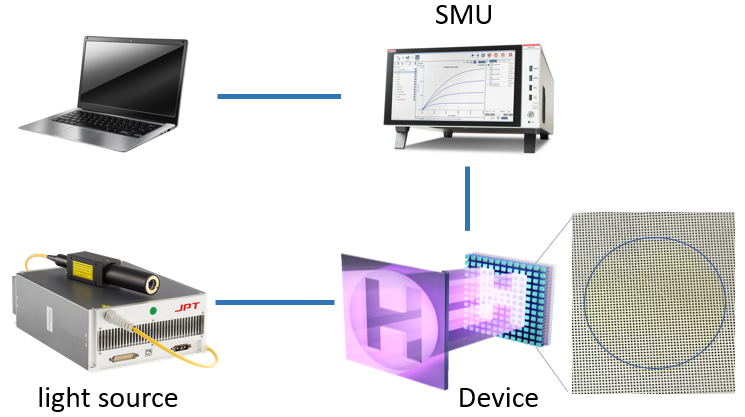


**Fig. S20** The schematic of the image sensing analysis system

**Table 1** Summary of key parameters of self-powered flexible and similar material structured photodetectors in this work and previous reports

| **Description** | **Flexibility** | **λ (nm)** | ***R* (A/W)** | ***D** (Jones)** | ***τ*_r_/*τ*_d_** | **Refs.** |
| --- | --- | --- | --- | --- | --- | --- |
| GaN NRAs/MoS_2_  /PEDOT:PSS | Yes | 365 | 1.47~2.47 | 1.2×10^11^~  2.6×10^11^ | 54/71~  40/45µs | This work |
| GaN NRAs/ZnO film | Yes | 325 | 2.82 | 6.82×10^13^ | 6.9/6.4ms | [S1] |
| 1D GaAs/2D WSe_2_ | Yes | 532 | 0.51 | 6.0×10^11^ | NA | [S2] |
| 1D Se/2D InSe | Yes | 460 | 0.11 | 5.6×10^11^ | 30/37ms | [S3] |
| 2D Ga_2_O_3_/2D PbI_2_ | Yes | 405 | 0.105 | 3.85×10^11^ | 2/3ms | [S4] |
| PbI_2_ Nanoplates | Yes | 405 | 0.65 | 0.95×10^13^ | 2/3ms | [S5] |
| 2D MoS_2_/Au | Yes | 450 | 0.431 | 2.54×10^8^ | 40/40ms | [S6] |
| Multilayer γ-InSe | Yes | 400 | 0.824 | 1.7×10^12^ | 20/10µs | [S7] |
| Cs_3_Cu_2_I_5_/PEDOT:PSS | Yes | 275 | 0.17 | 1.0×10^13^ | 177/195µs | [S8] |
| PEDOT:PSS/TiO_2_/ZnO | Yes | 325 | 1.257 | 2.9×10^13^ | 0.52/0.87s | [S9] |
| FA_0.8_PEA_0.2_SnI_3_ | Yes | 630 | 0.262 | 2.3×10^11^ | 25/42µs | [S10] |
| MoS_2_/GaN | No | 365 | 0.015 | 4.7×10^13^ | 4/8ms | [S11] |
| MoS_2_/GaN | No | 532 | 0.13 | 3.8×10^10^ | 18/123ms | [S12] |
| GaN/PEDOT:PSS | No | 365 | 0.96 | 5.7×10^12^ | 60/124ms | [S13] |

**Supplementary References**

1. Y. Peng, J. Lu, X. Wang, W. Ma, M. Que, et al., Self-powered high-performance flexible GaN/ZnO heterostructure UV photodetectors with piezo-phototronic effect enhanced photoresponse. Nano Energy **94**, 106945 (2022). <https://doi.org/10.1016/j.nanoen.2022.106945>
2. X. Chen, B. Jiang, D. Wang, G. Li, H. Wang, et al., Gate-tunable the interface properties of GaAs-WSe_2_ (1D-2D) vdWs heterojunction for high-responsivity, self-powered photodetector. Appl. Phys. Lett. **118**, 041102 (2021). <https://doi.org/10.1063/5.0035275>
3. H. Shang, H. Chen, M. Dai, Y. Hu, F. Gao, et al., A Mixed-dimensional 1D Se-2D InSe van der Waals Heterojunction for High Responsivity Self-powered Photodetectors. Nanoscale Horiz. **5**, 564-572 (2020). <https://doi.org/10.1039/c9nh00705a>
4. J. Zhang, F. Liu, D. Liu, Y. Yin, M. Wang, et al., Toward Smart Flexible Self-powered Near-UV Photodetector of Amorphous Ga_2_O_3_ Nanosheet. Mater. Today Phys. **31**, 100997 (2023). <https://doi.org/10.1016/j.mtphys.2023.100997>
5. D. Liu, R. Chen, F. Liu, J. Zhang, X. Zhuang, et al., Flexible Omnidirectional Self-powered Photodetectors Enabled by Solution-processed Two-dimensional Layered PbI_2_ Nanoplates. ACS Appl. Mater. Inter. **14**, 46748-46755 (2022). <https://doi.org/10.1021/acsami.2c13373>
6. X. Tang, S. Wang, Y. Liang, D. Bai, J. Xu, et al., High-performance, Self-powered Flexible MoS_2_ Photodetectors with Asymmetric van der Waals Gaps. PCCP **24**, 7323-7330 (2022). <https://doi.org/10.1039/d1cp05602f>
7. M. Dai, H. Chen, F. Wang, Y. Hu, S. Wei, et al., Robust Piezo-phototronic Effect in Multilayer γ-InSe for High-performance Self-powered Flexible Photodetectors. Acs Nano **13**, 7291-7299 (2019). <https://doi.org/10.1021/acsnano.9b03278>
8. T.-L. Shen, Y.-W. Chu, Y.-K. Liao, W.-Y. Lee, H.-C. Kuo, et al., Ultrahigh-performance Self-powered Flexible Photodetector Driven From Photogating, Piezo-phototronic, and Ferroelectric Effects. Adv. Opt. Mater. **8**, 1901334 (2020). <https://doi.org/10.1002/adom.201901334>
9. Y. Cheng, C. Li, J. Li, T. Li, Y. Sun, et al., Highly-sensitive, flexible, and self-powered UV photodetectors based on perovskites Cs_3_Cu_2_I_5_/PEDOT:PSS heterostructure. IEEE Electron Device Lett. **43**, 2137-2140 (2022). <https://doi.org/10.1109/led.2022.3218647>
10. M. He, Z. Xu, C. Zhao, Y. Gao, K. Ke, et al., Sn-based self-powered ultrafast perovskite photodetectors with highly crystalline order for flexible imaging applications. Adv. Funct. Mater. **33**, 2300282 (2023). <https://doi.org/10.1002/adfm.202300282>
11. T. J. Lin, W. L. Wang, Polarization-sensitive self-powered MoS_2_/a-GaN heterojunction photodetectors for ultraviolet polarized imaging. Appl. Phys. Lett. **124**, 252102 (2024). <https://doi.org/10.1063/5.0215741>
12. B. W. Liang, W. H. Chang, C. S. Huang, Y. J. Huang, J. H. Chen, et al., Self-powered broadband photodetection enabled by facile CVD-grown MoS_2_/GaN heterostructures. Nanoscale **15**, 18233-18240 (2023). <https://doi.org/10.1039/d3nr03877g>
13. S. Li, Z. Liu, M. L. Zhang, L. L. Yang, Y. F. Guo, et al., High-performance self-powered GaN/PEDOT:PSS hybrid heterojunction UV photodetector for optical communication. Sci. China Tech. Sci. **67**, 608-615 (2024). <https://doi.org/10.1007/s11431-023-2501-5>
